# Supplementary material for: Protein purification with light via a genetically encoded azobenzene side chain
Source: Nat Commun. 2024 Dec 18;15:10693. doi: 10.1038/s41467-024-55212-y (PMC11655525; doi:10.1038/s41467-024-55212-y)
Supplement: Supplementary file 1 — Supplementary Information [file 41467_2024_55212_MOESM1_ESM.pdf]

Supplementary Information

Protein purification with light via a genetically encoded azobenzene side chain

Peter Mayrhofer[1], Markus R. Anneser[1], Kristina Schira[1], Carina A. Sommer[1], Ina Theobald[1], Martin Schlapschy[1], Stefan Achatz[1], Arne Skerra[1]*

[1]Chair of Biological Chemistry, School of Life Sciences, Technical University of Munich, 85354 Freising, Germany

*Corresponding author:
Prof. Dr. Arne Skerra; phone: +49 8161 71 4351; fax: +49 8161 71 4352; e-mail: skerra@tum.de

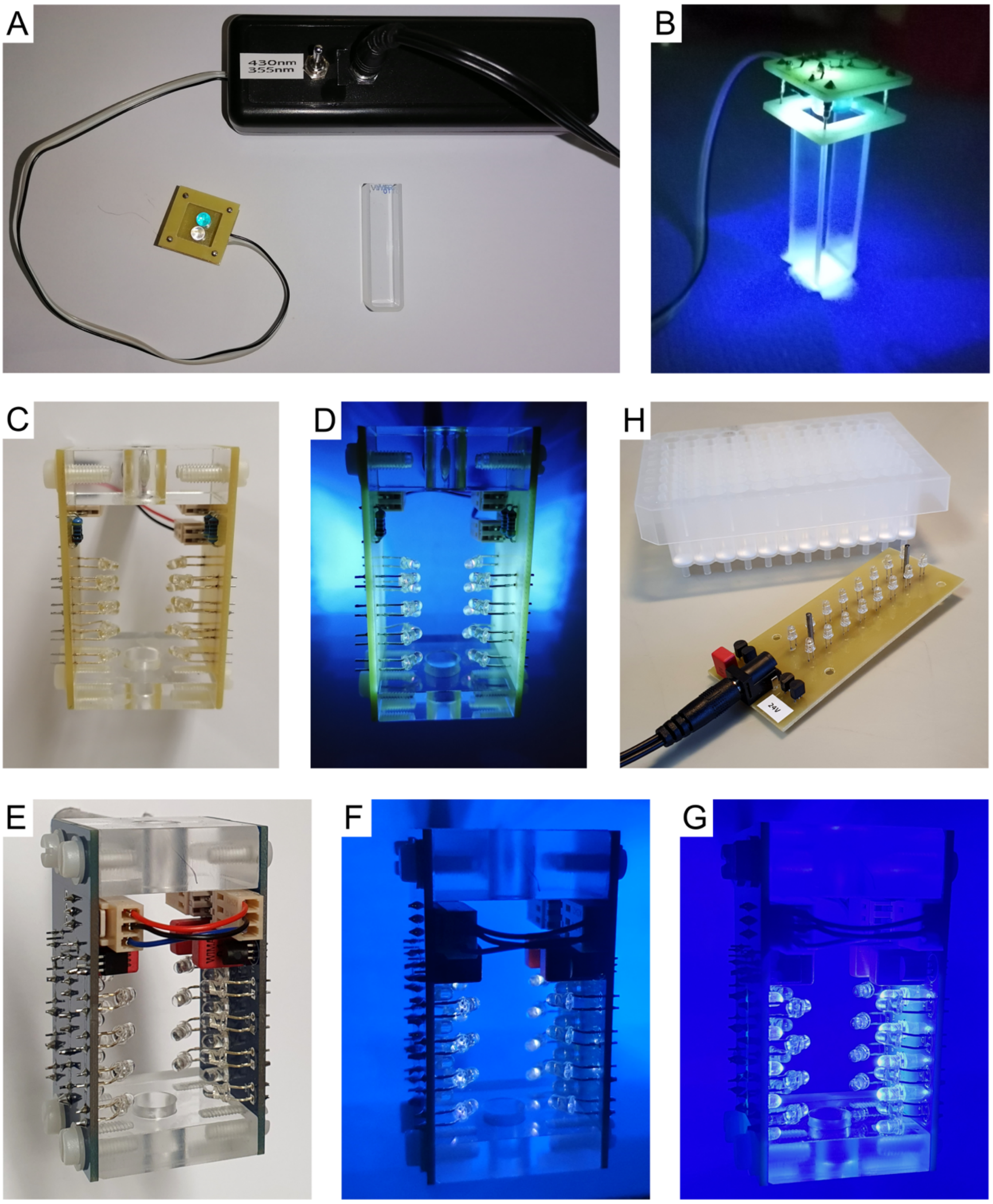

Suppl. Fig. S1. Home-made devices for the photo-induced *trans*/*cis*-isomerization of Pap. (A, B) LED device for the irradiation at two wavelengths, 355 nm (1.2-2.4 mW LED) or 430 nm (27 mW LED), of samples in a 4 mL (1x1 cm^2) quartz cuvette from the top for UV/Vis measurements in a photometer. (C, D) Setup for the illumination of the α-CD chromatography matrix (1 mL bed volume in a Pierce disposable 2 mL column) at 355 nm using in total up to 16 LEDs (4 vertical rows with each four 1.2-2.4 mW LEDs; 2 rows with each four LEDs in an earlier version). (E to G) Extended setup that allows illumination of the column either at 355 nm (F; 4x4 LEDs, each 1.2-2.4 mW, front and back rows) or at 430 nm (G; 2x4 LEDs, each 27 mW, middle rows). (H) Setup for the illumination of two rows of a 96-well receiver plate (one 355 nm LED, 1.2-2.4 mW, per well).

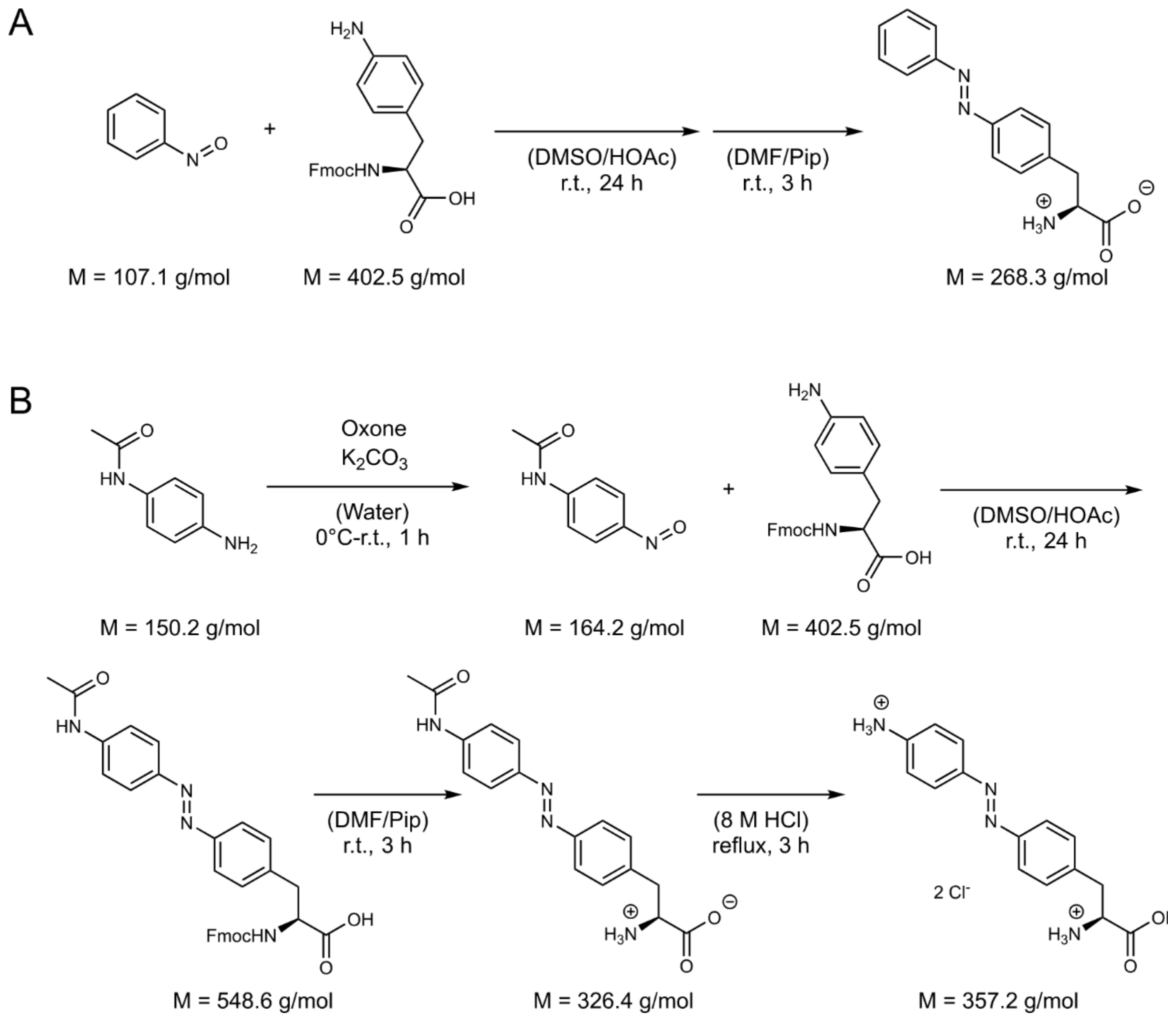

Suppl. Fig. S2. Chemical synthesis schemes for Pap and NH_2-Pap.

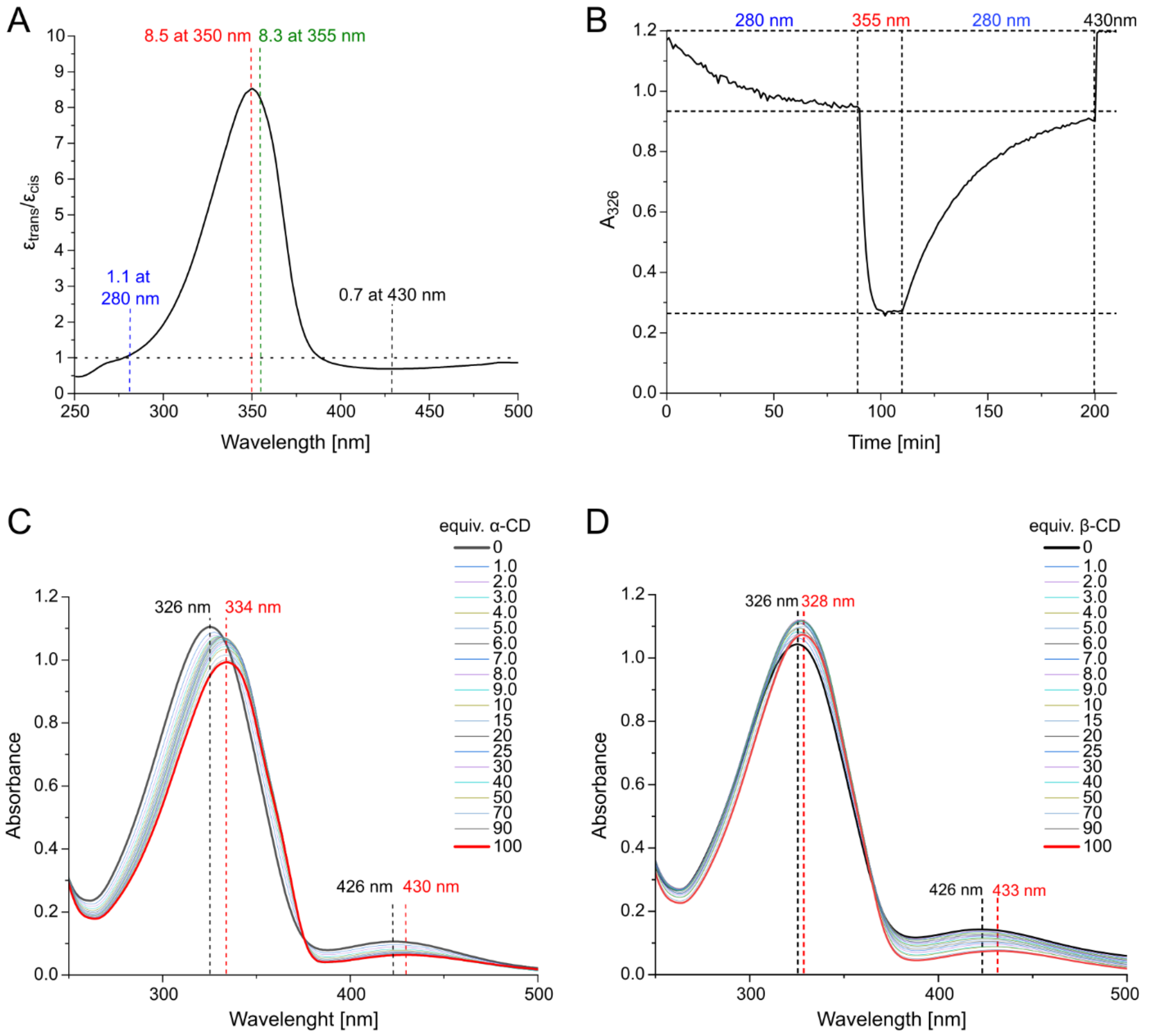

Suppl. Fig. S3. Spectral changes upon transition between the *trans*- and *cis*-configurations of Pap and their complex formation with CDs. (A) Ratio $\varepsilon_{trans}/\varepsilon_{cis}$ of a 50 µM solution of Pap in 100 mM Tris/HCl pH 8.0 (cf. Fig. 2D) plotted *versus* the wavelength. A prominent maximum at 350 nm indicates the optimal wavelength for photo-induced *trans*-to-*cis* isomerization with a theoretical ratio of 8.5. (B) Choice of the available LED wavelength governs the experimentally achievable *cis*/*trans* isomer ratio. For example, when irradiating *trans*-Pap at 280 nm UV light, only a low degree of *cis*-isomerization takes place at the PSS, whereas upon subsequent irradiation at 355 nm the *cis*-configuration is almost quantitatively adopted in a short time. Conversely, subsequent irradiation at 280 nm again results in a mixed *trans*-/*cis*-equilibrium, which shifts almost completely to the *trans*-state if exposed to visible light at 430 nm. (C) Smoothed absorption spectra from a typical titration experiment of 50 µM *trans*-PAP in 2 mL 100 mM Tris/HCl pH 8.0 (cf. Fig. 2I) with 50 mM α-CD in the same buffer, revealing shifts in λ_{max} and amplitude for both bands (no correction for the dilution effect was applied). (D) Similar absorption spectra from a titration experiment of 50 µM *trans*-Pap (cf. Fig. 2K) with 50 mM β-CD. Contrasting to the decreasing amplitude in the case of α-CD, a slightly increasing amplitude (if also considering the dilution effect) was observed for β-CD. Similar deviations have been observed for other azobenzene derivatives when forming complexes with different CDs[1]. This is likely explained by distinct intermolecular interactions within the corresponding host-guest complexes, in line with the fact that β-CD can also accommodate the more bulky *cis*-isomer whereas α-CD cannot.

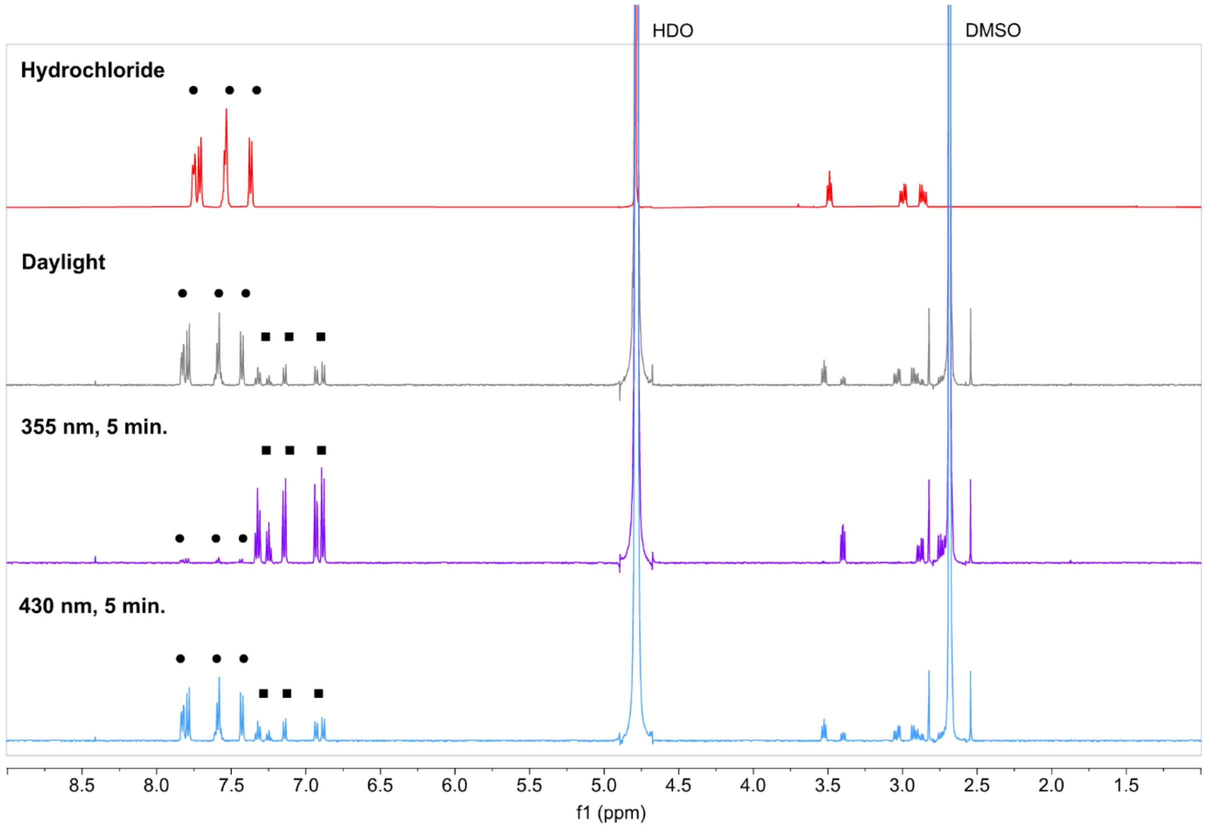

Suppl. Fig. S4. ^{1}**H-NMR spectra of a ~4.5 mM Pap solution in** D_2O **(with 50 mM DMSO as internal standard; 2.61 ppm):**
(i) measured for the Pap hydrochloride, solid dissolved with addition of NaOD (no DMSO), in the dark,
(ii) measured for a solution of the synthesized Pap after 2 h incubation at 25 °C under daylight,
(iii) after irradiation at 355 nm for 5 min and
(iv) after irradiation at 430 nm for 5 min.
Note that in particular the peaks in the aromatic region are considerably shifted to higher field upon change from the *trans*- (•) to the *cis*-configuration (▪).

trans-Pap: ^{1}H-NMR (500 MHz, D_2O): δ = 7.76 (dd, J = 7.3 Hz, 2H, CH), 7.72 (d, J = 8.0 Hz, 2H, CH), 7.52 (m, 3H, CH), 7.39 (d, J = 8.0 Hz, 2H, CH), 3.46 (dd, 1H, αCH), 2.97 (dd, 1H, CH_2) 2.85 (dd, 1H, CH_2) ppm

cis-Pap: ^{1}H-NMR (500 MHz, D_2O): δ = 7.25 (d, J = 7.3 Hz, 2H, CH), 7.18 (d, J = 8.0 Hz, 2H, CH), 7.08 (m, 3H, CH),6.82 (d, J = 8.0 Hz, 2H, CH), 6.86 (d, J = 8.0 Hz, 2H, CH), 3.33 (dd, 1H, αCH), 2.81 (dd, 1H, CH_2) 2.67 (dd, 1H, CH_2) ppm

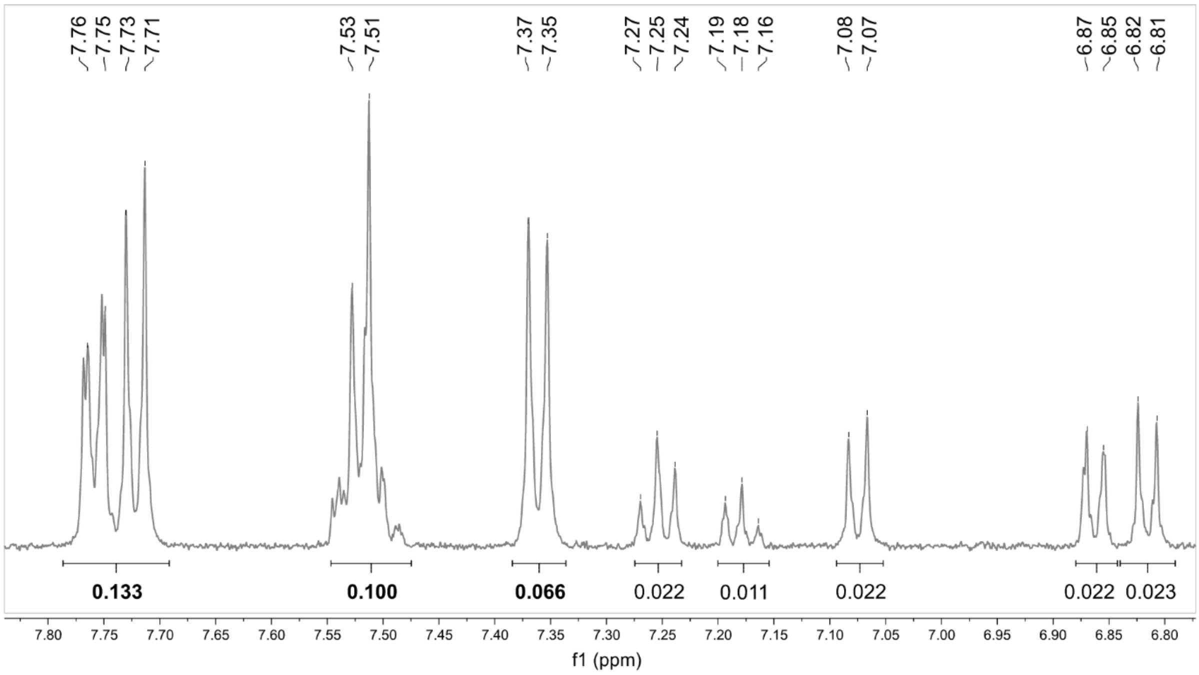

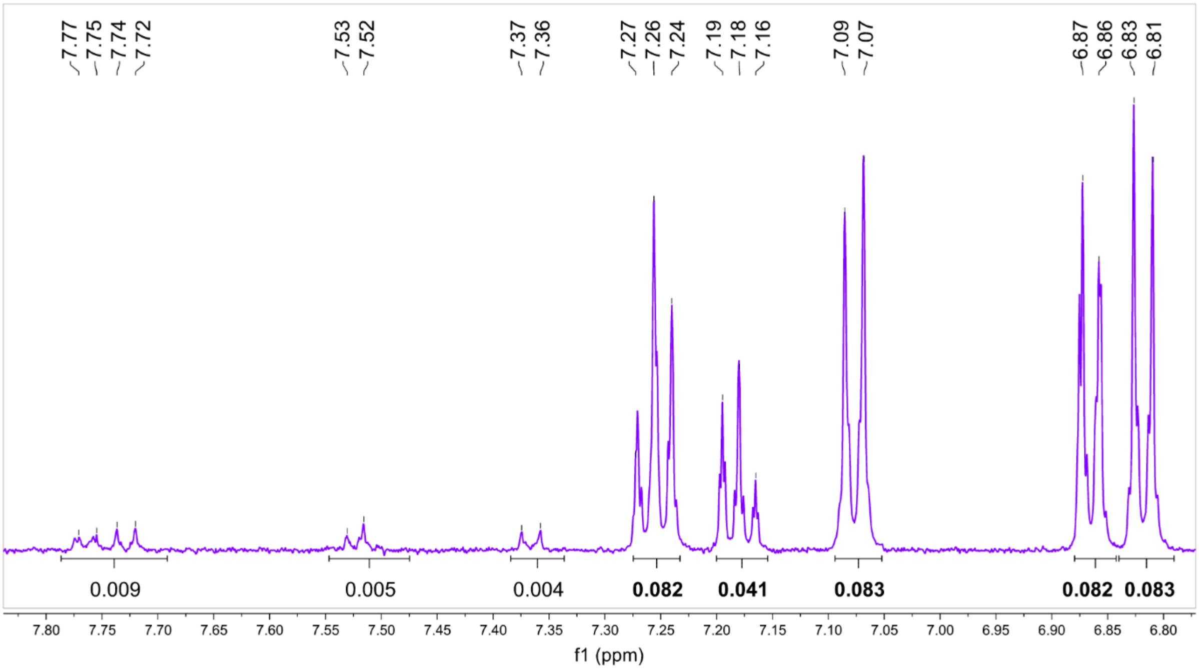

Suppl. Fig. S5. Aromatic region of a ~4.5 mM Pap solution integrated relative to the DMSO standard in D_2O (cf. Suppl. Fig. S4). Top, after 2 h at 25 °C under daylight predominantly the *trans*-state (bold integral values) is generated (for quantification, see Suppl. Fig. S6); bottom, irradiation at 355 nm for 5 min yields almost pure *cis*-isomer (bold integral values).

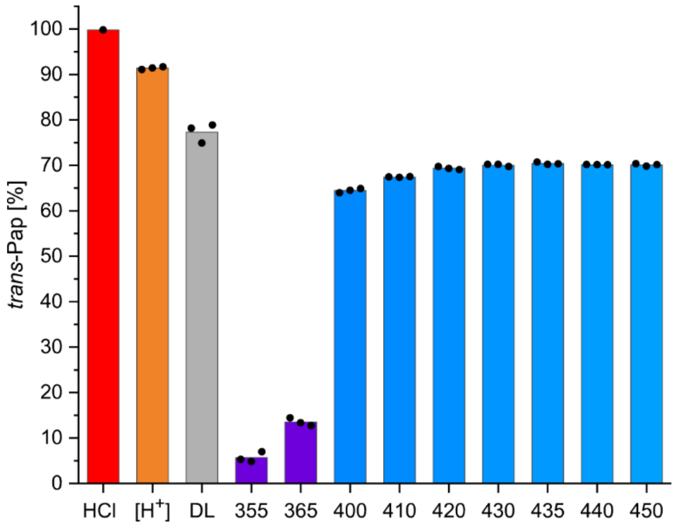

Light condition	*cis*-Pap [%]	*trans*-Pap [%]	SD (N=3)
Dark, hydrochloride	0.2	99.8	–
Dark, [H+] (24 h)	8.6	91.4	0.3
Daylight (2 h)	22.7	77.3	2.1
355 nm	94.3	5.7	1.1
365 nm	86.5	13.5	0.9
400 nm	35.5	64.5	0.5
410 nm	32.6	67.4	0.1
420 nm	30.6	69.4	0.4
430 nm	30.0	70.0	0.3
435 nm	29.6	70.4	0.3
440 nm	30.0	70.0	0.1
450 nm	29.9	70.1	0.3

Suppl. Fig. S6. Proportion of *trans*-Pap determined from ^{1}H-NMR spectra (at 25 °C) of a ~4.5 mM Pap solution in D_2O (with 50 mM DMSO as internal standard) after illumination at the indicated wavelength for 5 min (measured as triplicates; data see the Table underneath). [H^+] corresponds to Pap after thermal relaxation to the *trans*-state, which was enforced by protonation of the azo-group via transient acidification[2]. To this end, 2.5 mg Pap and 5 µL DMSO were mixed with 1.5 mL D_2O and 30 µL conc. HCl was added, resulting in a clear orange solution which was applied to three NMR tubes (500 µL each). After storage for 24 h at room temperature in the dark the NMR spectra were collected. Note that the *cis*-state of Pap is almost quantitatively populated after illumination with LED UV light at 355 nm, but significantly less at a wavelength of 365 nm (as it has been commonly used in other studies with this ncAA).

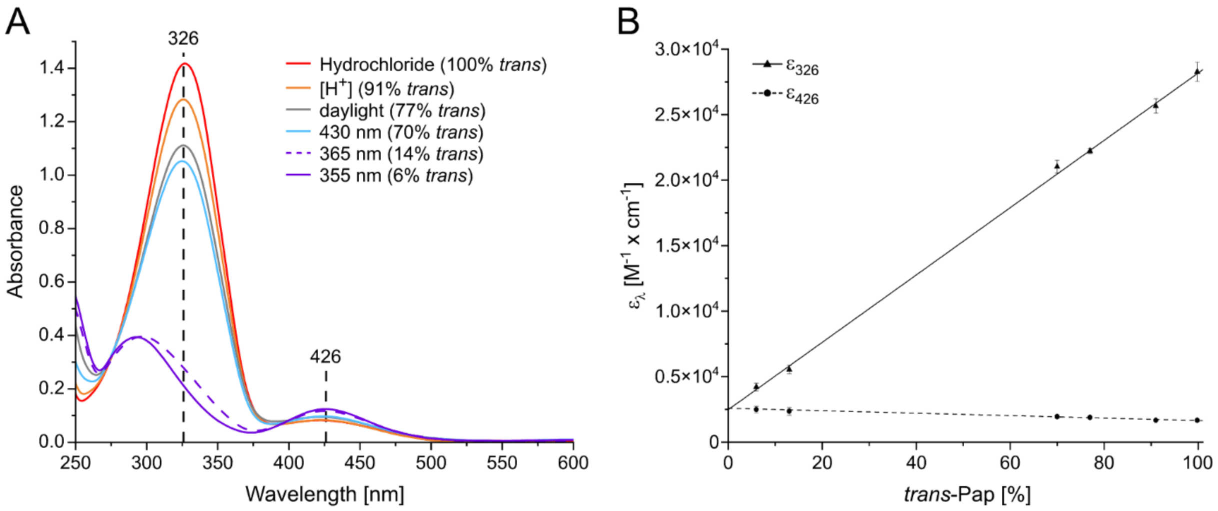

Suppl. Fig. S7. Detailed analysis of the UV-Vis spectra of Pap in aqueous solution at different PSS. (A) A 50 µM solution of Pap in 100 mM Tris/HCl pH 8.0 was either exposed to daylight for 2 h or illuminated from the top (see Suppl. Fig. S1) with LED light at 430 nm for 5 min, at 355 nm for 30 min or at 365 nm for 30 min (N=3). To measure the spectrum of Pap in the full *trans*-state, the freshly dissolved hydrochloride was applied or, alternatively, almost complete thermal relaxation was induced by protonation of the azo-group via transient acidification[2]. To this end, 500 µL of a 5 mM stock solution of Pap in 10 mM NaOH was mixed with 500 µL 1 M HCl and 1.5 mL water. The solution was shielded from light by wrapping in aluminum foil and incubated for 1 d at 20 °C. Then, 100 µL of this solution was added to 1.9 mL 100 mM Tris/HCl pH 8.0 in a quartz cuvette (1x1 cm^2) and stirred with a small magnetic bar for 1 min in the dark, directly followed by measurement of the spectrum. All spectra were corrected by subtraction of the corresponding buffer blank; wavelengths of the absorption maxima are indicated. (B) The mean extinction coefficients (N=3) at the two characteristic wavelengths, ε_{326} (*trans*-state) and ε_{426} (*cis*/*trans*-state), measured at the different PSS shown in (A) were fitted by linear regression *versus* the respective content of the *trans*-isomer of Pap as quantified by ^{1}H-NMR (see Suppl. Fig. S6), yielding the following relations ([*t*Pap] corresponds to the proportion of *trans*-Pap in %):

$$\epsilon_\lambda = \varepsilon_\lambda^0 + \epsilon_\lambda^\% \times [t\mathrm{Pap}]$$

λ [nm]	ε_λ^0 [M^{-1} cm^{-1}]	$\varepsilon_\lambda^\%$ [M^{-1} cm^{-1} $\%^{-1}$]
326	2480 ± 245	257.0 ± 3.9
426	2585 ± 175	-9.37 ± 2.14

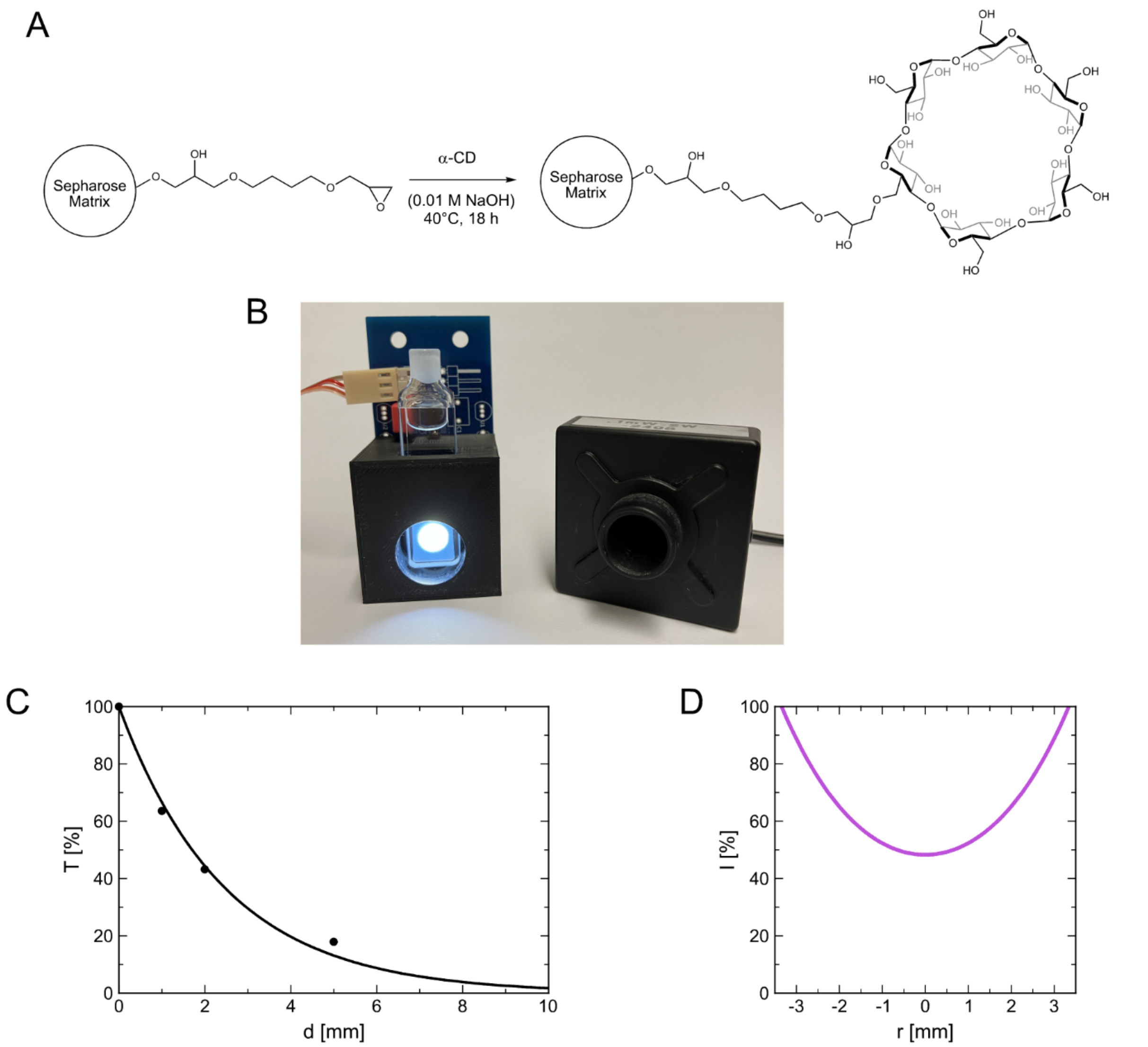

Suppl. Fig. S8. Synthesis and translucent properties of an α-CD affinity column. (A) Chemical synthesis of the α-CD-conjugated Sepharose matrix. The primary hydroxyl groups at the C6-position of the glucose residue are more likely to react with the epoxide. (B) Setup to assess the light transmission through layers of the α-CD chromatography matrix with varied thickness. A suspension of the matrix in 100 mM Tris/HCl pH 8.0 buffer was applied to a quartz cuvette (1 cm x 1 / 2 / 5 mm) and settled for 1 d. The cuvette was placed in a black holder, made by 3D-printing, and illuminated from the back with a white-light emitting LED (B3B-440-LBS, 3 mm diameter, 10° viewing half angle, 95 mW, prominent emission peak at ~470 nm; Roithner LaserTechnik) through a circular aperture (6 mm diameter). The energy of the transmitted light was measured using a FieldBest Optical Power Meter (Shenzhenshi Tianzhongtian Trading, Guangdong, China) with a circular opening (12 mm diameter) placed directly at the front of the cuvette. The same cuvette filled with buffer was used as blank (T = 100 %). (C) The measured transmission, $T = I/I_0$, was correlated with the thickness d of the affinity matrix layer according to the physical law of light attenuation, which describes the reduction in light intensity due to absorption or scattering of photons in a medium: $T = e^{-\mu d}$. Curve fit resulted in an attenuation coefficient μ = 0,41 ± 0.03 mm^{-1}, corresponding to a half-width of 1.7 mm (i.e., the thickness of the α-CD chromatography matrix leading to a reduction in transmission by 50 %). Note that the translucence also depends on the buffer composition as light scattering is governed by the different refractive indices of the particles in the chromatography matrix (solid phase) and the mobile phase. (D) Simulated profile of the light intensity (I) *versus* radius (r) for an α-CD affinity column with 7 mm inner diameter illuminated from both sides, as used for the chromatography experiments throughout this study.

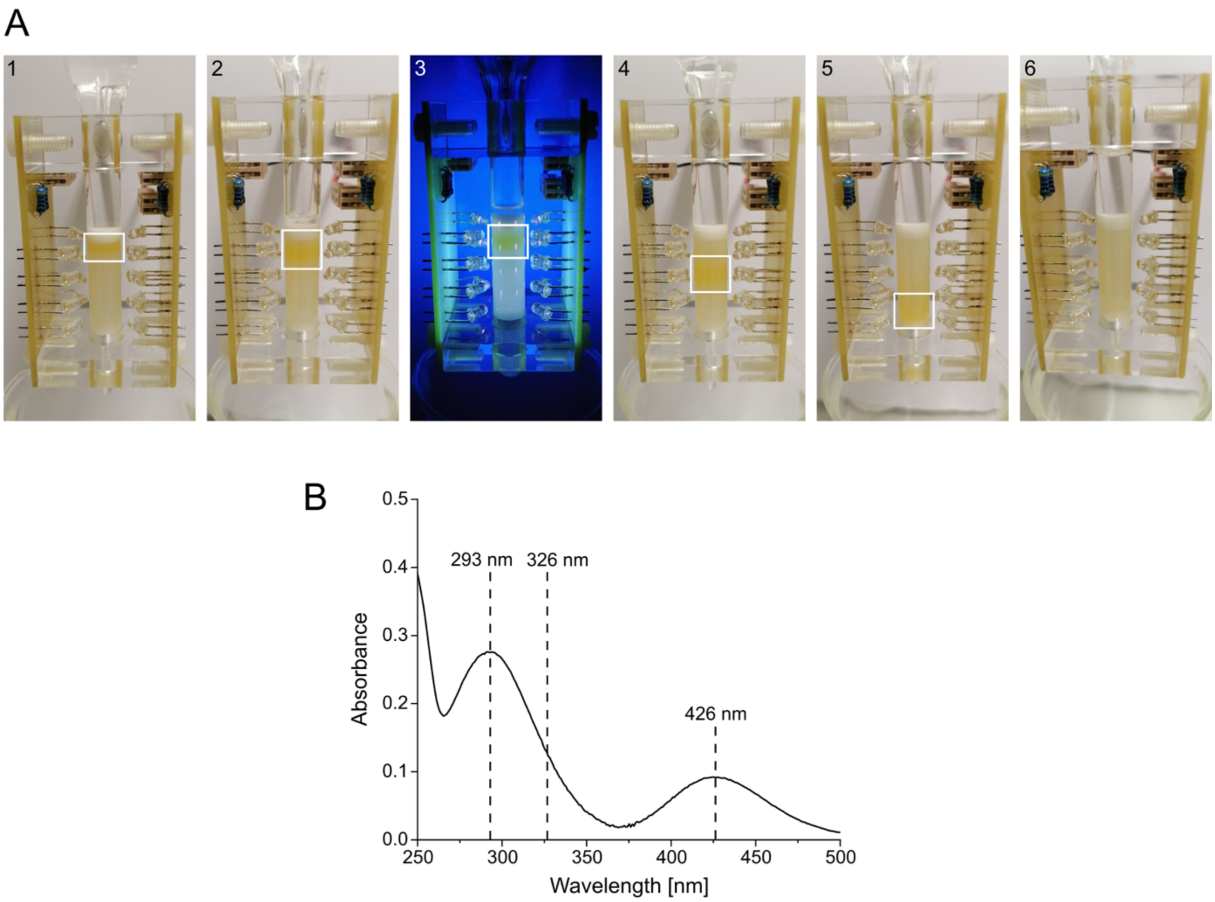

Suppl. Fig. S9. Light-controlled adsorption and desorption of Pap on/from a synthesized α-CD affinity column. (A) A light-controlled chromatography experiment with Pap. (**1**) 100 μL of 5 mM *trans*-Pap in 10 mM NaOH was loaded on the α-CD affinity column (1 mL bed volume). (**2**) The column was washed with 10 bed volumes of 100 mM Tris/HCl 8.0, 0.5 M NaCl; the yellow band corresponding to the adsorbed Pap is essentially retained at the top. (**3**) The column was illuminated at 355 nm for 10 min. (**4, 5, 6**) The column was washed with 0.5 mL buffer aliquots, leading to the complete elution of Pap. (B) UV-Vis spectrum immediately measured for the UV-eluted fraction (picture 6 in A) after 1:20 dilution with 100 mM Tris/HCl pH 8.0. Only the characteristic absorption bands at 293 and 426 nm of Pap in the *cis*-configuration are visible, whereas the absorption band at 326 nm typical for the *trans*-isomer is absent.

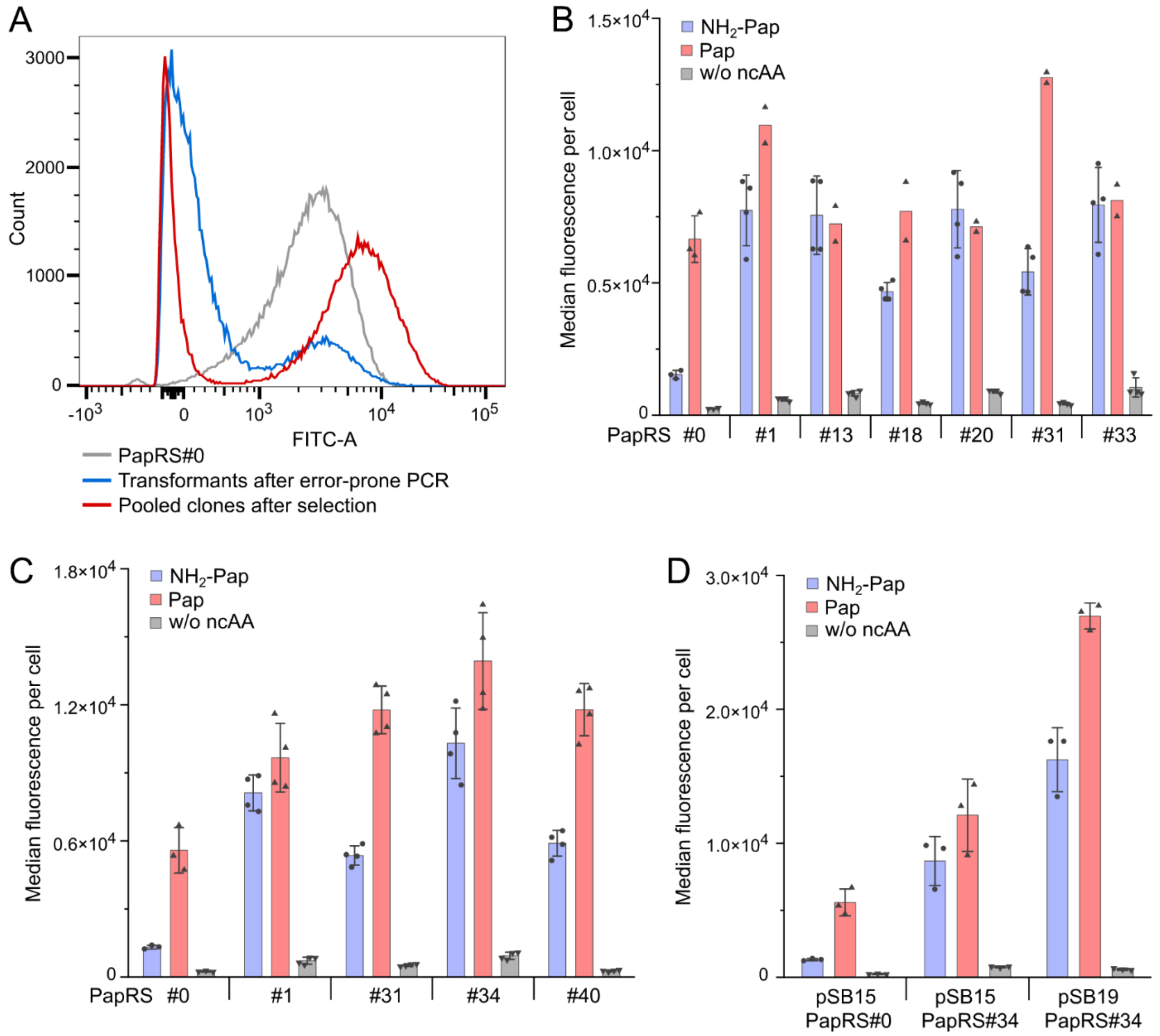

Suppl. Fig. S10. Directed evolution of PylRS to efficiently charge $tRNA^{Pyl}$ with Pap and/or NH_2-Pap. (A) Selection of a library generated by error-prone PCR of PapRS#0 for improved incorporation of the ncAA after bacterial growth in the presence of 1 mM NH_2-Pap. The histogram depicts the sfGFPa39 fluorescence (10^5 events) measured by FACS. The fluorescence of the clones obtained after the final selection cycle of this first directed evolution campaign (red) clearly increased compared to the initial variant PapRS#0 (grey) and to the pooled transformants directly obtained after the random mutagenesis (blue). (B) MFI of sfGFPa39 in the presence of 0.5 mM Pap (red), 0.5 mM NH_2-Pap (blue) or in the absence of a ncAA (grey) as measured by FACS (10^5 events) for several mutants resulting from the directed evolution of PapRS#0 (N=2–4 individual experiments). (C) MFI of sfGFPa39 measured by FACS as in (B) for several mutants obtained either from the directed evolution of PapRS#0 or by specific combination of beneficial mutations (N=4). (D) MFI of sfGFPa39 in the presence of 0.5 mM Pap, 0.5 mM NH_2-Pap or in the absence of a ncAA as determined by FACS (10^5 events) for the PylRS mutants PapRS#0 and PapRS#34 expressed from either the pSB15 or the pSB19 vector (N=3). Error bars in (B), (C) and (D) indicate standard deviation (for N≥3).

A

```
         190       200       210       220       230       240       250       260       270       280       290       300
         .         .         .         .         .         .         .         .         .         .         .         .
PapRS#0  LTRSQTDRLEVLLNPKDEISLNSGKPFRELESELLSRRKKDLQQIYAEERENYLGKLEREITRFFVDRGFLEIKSPILIPLEYIERMGIDNDTELSKQIFRVDKNFCLRPMLTPNLYNYS
PapRS#1  ..........A....................R........................................................................................
PapRS#13 .............H.....N...........................................................S........................................
PapRS#18 ...............................R.........................................................................L........S.....
PapRS#20 ...................................R........M............................................................L..............
PapRS#31 ........................................................................................................................
PapRS#33 ...............................R........................................................................................
PapRS#34 .........................................................................................................L........S.....
PapRS#40 ........................................................................................................................

         310       320       330       340       350       360       370       380       390       400       410       420
         .         .         .         .         .         .         .         .         .         .         .         .
PapRS#0  RKLDRALPDPIKIFEIGPCYRKESDGKEHLEEFTMLVFGQMGSGCTRENLESIITDFLNHLGIDFKIVGDSCMVFGDTLDVMHGDLELSSAVVGPIPLDREWGIDKPWIGAGFGLERLLK
PapRS#1  ....................................A.......................M...........................................................
PapRS#13 ....................................A...................................................................N...............
PapRS#18 ........................................................................................................................
PapRS#20 ....................................A...................................................................................
PapRS#31 ........................................................................................................................
PapRS#33 ....................................A...............................D...................................................
PapRS#34 ....................................A...................................................................................
PapRS#40 ....................................A...................................................................................
```

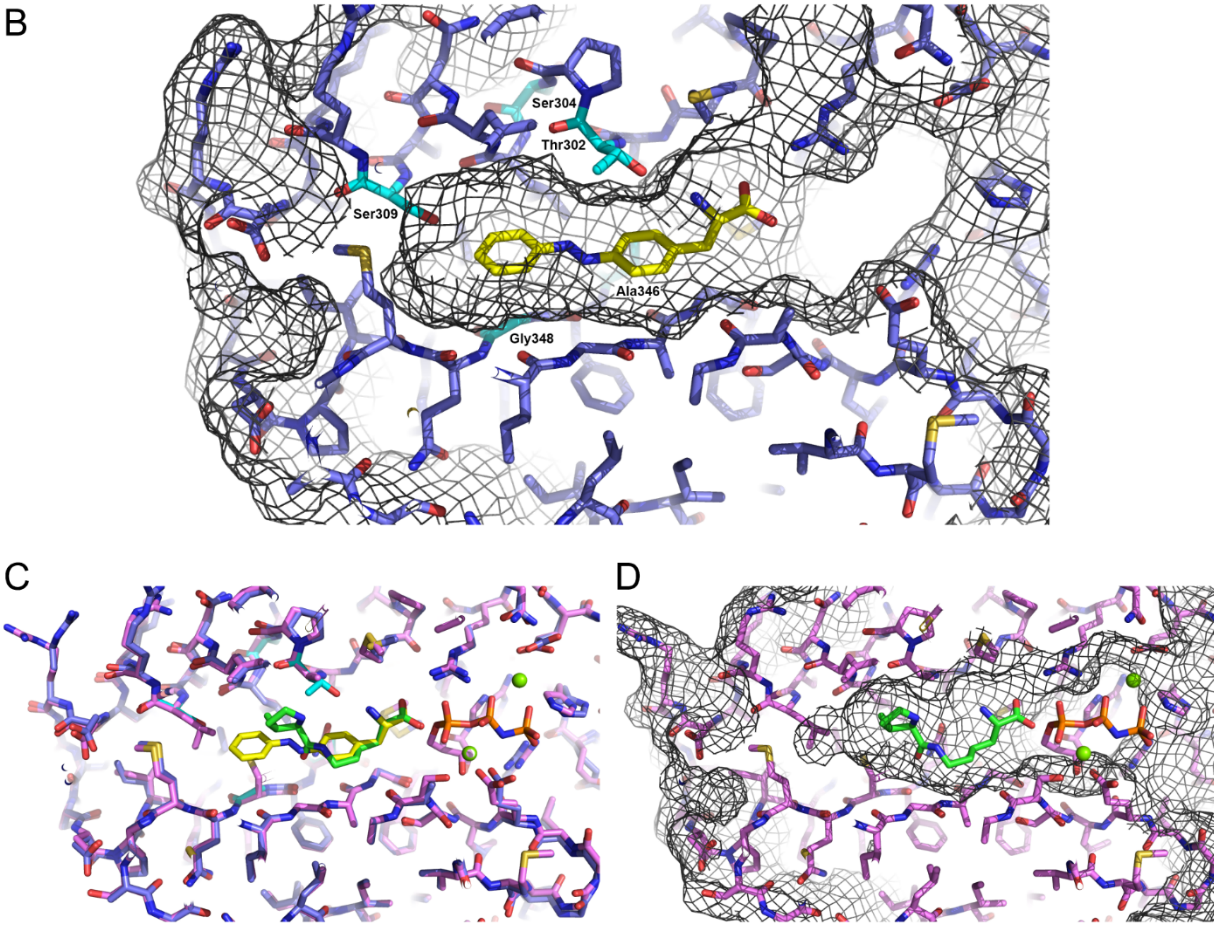

Suppl. Fig. S11. Sequence and structural properties of PapRS#34. (A) Amino acid sequence alignment of relevant PylRS mutants isolated in the course of the directed evolution of PylRS to efficiently charge $tRNA^{Pyl}$ with Pap and/or NH_2-Pap (see Suppl. Fig. S10). Mutations discussed in the text are highlighted bold. (B) Molecular model of PapRS#34 generated using AlphaFold 2.3.2[3]. The accessible protein surface is shown with a mesh and *trans*-Pap (carbon yellow) was modelled into the amino acid substrate pocket. Visible mutated residues are highlighted in light blue and labeled. (C) Superposition of the model coordinates of PapRS#34 onto the published crystal structure of wild-type PylRS with bound L-pyrrolysine (carbon green) and a non-hydrolyzable ATP analog[4] (PDB ID: 2ZCE). (D) Similar representation with accessible protein surface for wild-type PylRS as in (B), thus indicating a more straight and elongated substrate pocket for the engineered enzyme, PapRS#34 (see B).

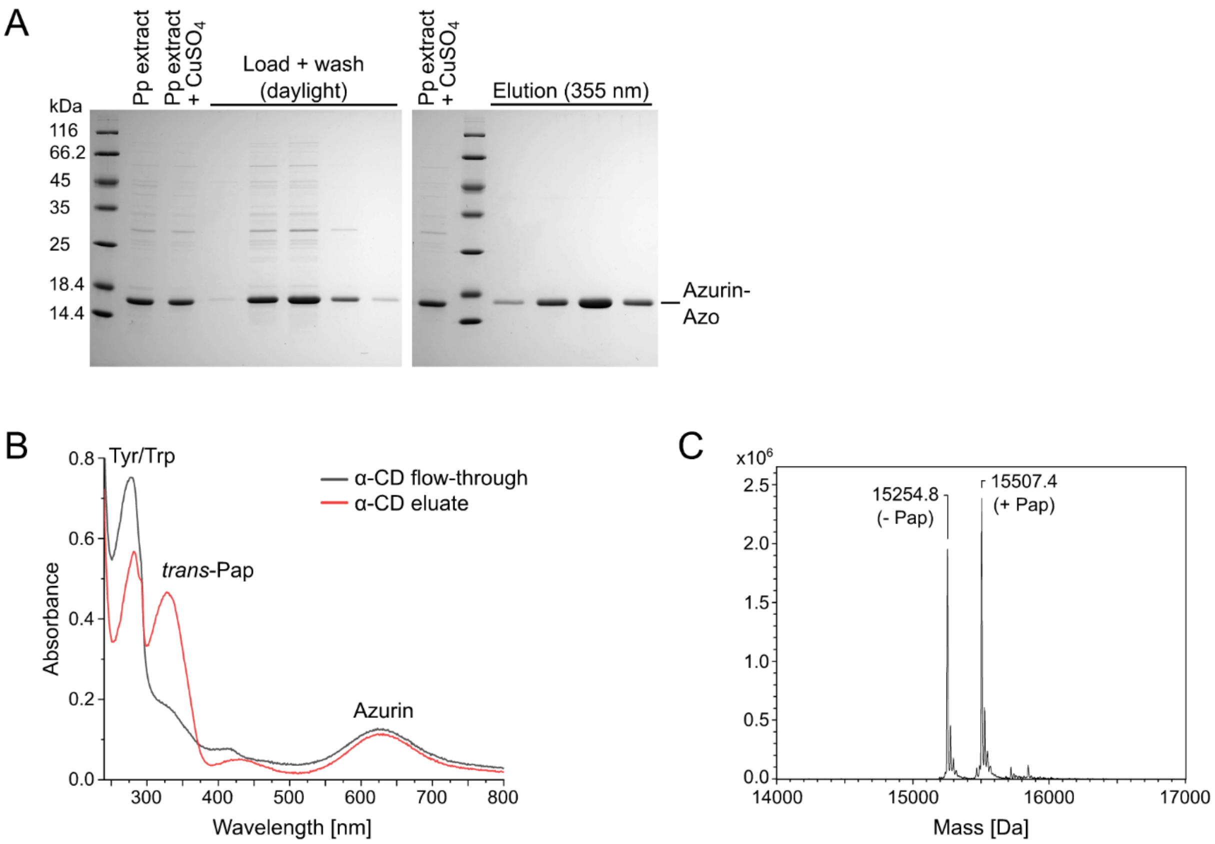

Suppl. Fig. S12. Purification of Azurin-*Strep*-GG-Pap from the bacterial periplasmic extract via α-CD affinity chromatography and incomplete stop codon suppression. (A) The periplasmic cell fraction of NEBExpress(lowRF1) containing the secreted POI was directly applied to the α-CD affinity column (1 mL bed volume), followed by washing with 3 bed volumes of chromatography buffer A (under daylight) until all host cell proteins were removed. While a large portion of the blue POI remained bound at the top of the column during this step, some unbound Azurin also appeared in the flow-through. However, upon exposure to 355 nm UV light, the remaining bound Azurin-*Strep*-GG-Pap specifically eluted from the column without contaminating host cell proteins. (B) Absorption spectrum of the flow-through and the light-induced eluate fractions, following exposure to daylight (in order to revert Pap to the *trans*-configuration). (*trans*)-Pap absorption was largely absent in the unbound Azurin fraction compared to the UV eluate, indicating a partial lack of the C-terminal Pap residue in the biosynthetic protein. (C) ESI-MS analysis of Azurin-*Strep*-GG-Pap purified via Strep-Tactin affinity chromatography. Incomplete amber codon suppression resulted in a mixture of the Azo-tagged POI (calc. 15506.5 Da) and untagged protein, which only lacks the C-terminal Pap residue (calc. 15256.2 Da) and cannot bind to the α-CD affinity column.

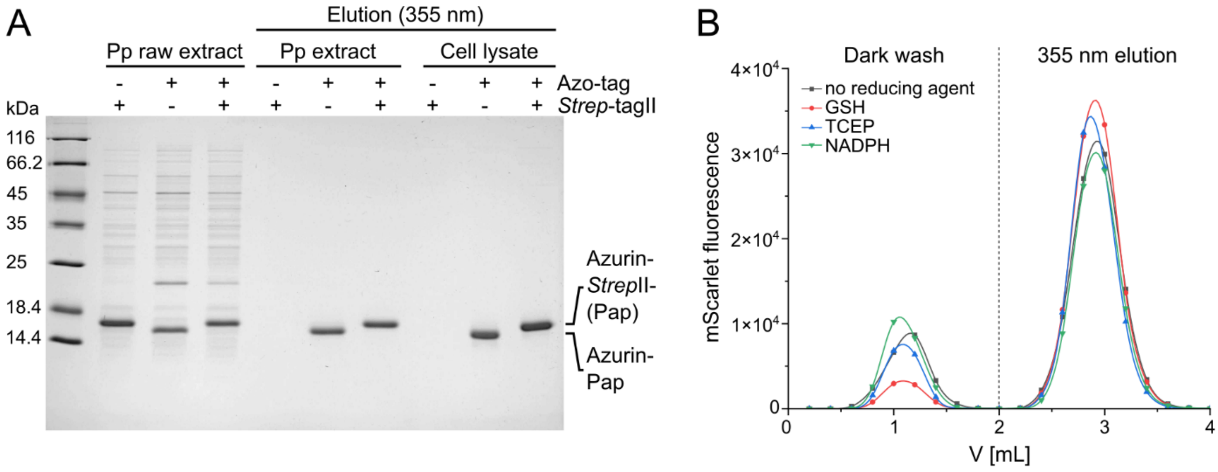

Suppl. Fig. S13. The negligible role of the *Strep*-tag II and stability of the Azo-tag *versus* biochemical reducing agents. (A) To investigate a potential influence of the *Strep*-tag II, which had been included for convenience, on the light-controlled α-CD affinity chromatography of Azo-tagged POIs, Azurin-*Strep*-GG-Pap, Azurin-GG-Pap and Azurin-*Strep*-GG (prepared by expression in the absence of the ncAA, thus lacking the Azo-tag) were purified from periplasmic (Pp) extracts as well as whole cell lysates of *E. coli*. While the expression levels of the different POI versions were similar, as indicated by the comparable band intensities in the SDS-PAGE samples of the Pp raw extracts (not shown for the whole cell lysates as there was not prominent band visible), the *Strep*-tag II neither led to a detectable interaction with the α-CD resin by itself nor contributed to the efficient light-controlled protein purification via the Azo-tag. (B) Effect of common biochemical reducing agents on the Azo-tag. Prior to the α-CD affinity chromatography, purified mScarlet-*Strep*-GG-Pap was incubated for 16 h at room temperature in the dark, either without a reducing agent (grey) or with 10 mM GSH (red), 5 mM TCEP (blue) or 10 mM NADPH (green). The performance of the resulting protein preparation in the subsequent light-controlled chromatography was analyzed via fluorescence measurement of 200 µl fractions (in total 2 mL washing with chromatography buffer B in the dark, followed by 2 mL elution with the same buffer) in a Synergy 2 microtiter plate reader. The chromatographic behavior was similar to other Azo-tagged POIs, with a small fraction (apparently corresponding to the proportion of *cis*-isomer generated during preceding steps performed under daylight, see Suppl. Fig. S14) in the flow-through and the majority specifically eluted upon exposure to 355 nm UV light. However, there was no relevant sign of diminished light-controlled elution of the Azo-tagged POI after incubation with one of the reducing agents, as would have been expected if the azo-group had been chemically reduced to a hydrazine or even cleaved (see Suppl. Fig. S17).

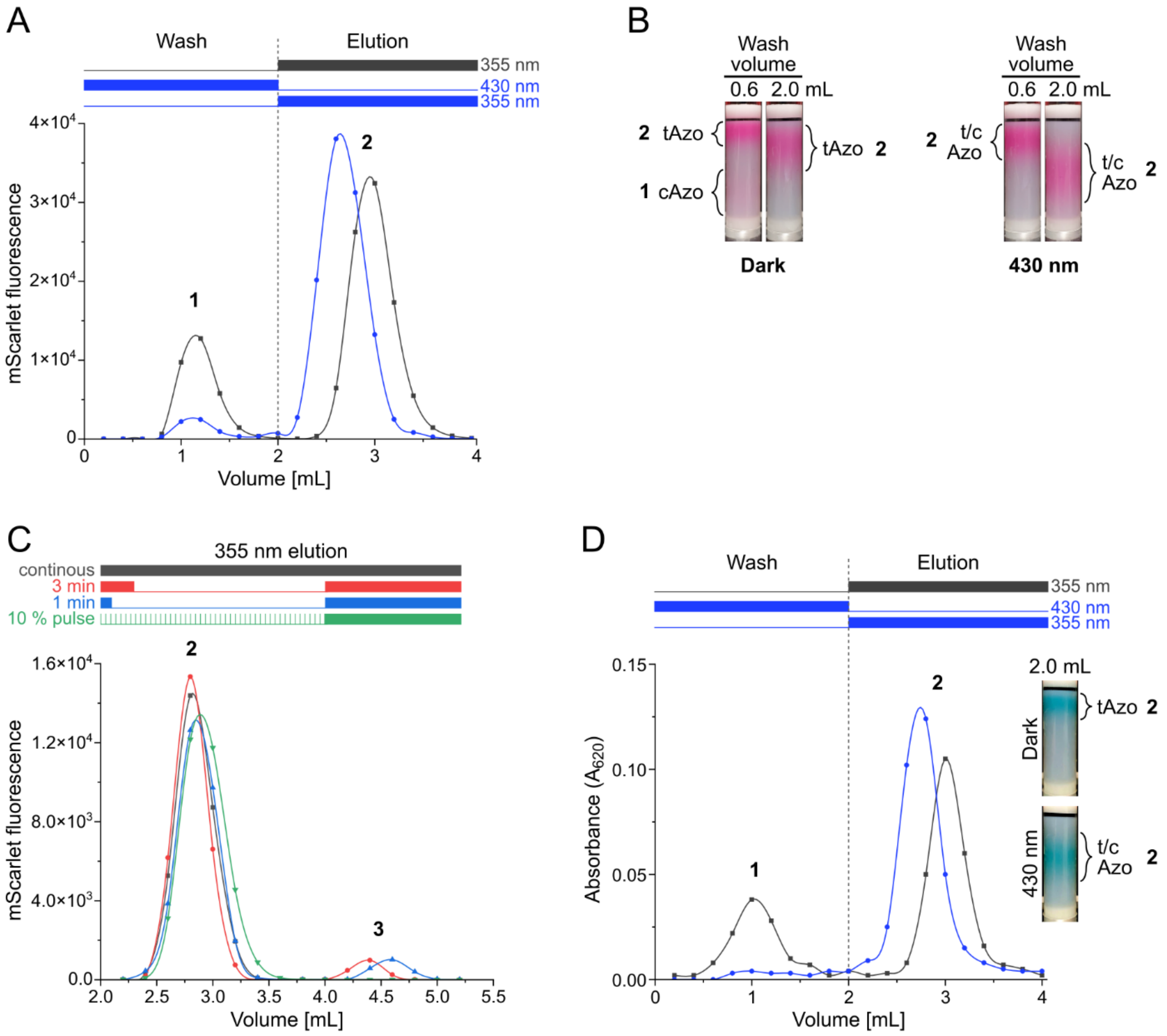

Suppl. Fig. S14. Influence of the mode and time of illumination during sample application and washing, or the UV-induced elution step, on the chromatographic performance of different Azo-tagged POIs. (A) α-CD affinity chromatography of mScarlet-*Strep*-GG-Pap using chromatography buffer B, analyzed via its characteristic fluorescence (λ_{Ex} = 530nm, λ_{Em} = 590 nm, measured with a Synergy 2 microtiter plate reader) in 200 µL fractions. The sample was applied and then immediately washed with buffer either in the dark lab (gray) or upon continuous exposure to 430 nm LED light (blue; cf. Suppl. Fig. S1G), each time followed by elution under 355 nm LED UV light. During washing in the dark, 25 % of the total fluorescent protein appeared in the flow-through (i.e., prior to the UV-induced elution, peak 1), corresponding to the proportion of the Pap *cis*-isomer in the PSS under daylight (see Suppl. Fig. S6), which remains fixed in the dark and does not interact with the α-CD groups (cf. Fig. 1). However, when washing was performed under continuous illumination at 430 nm, which induces the dynamical reequilibration of the photostationary mixture of *cis*- and *trans*-isomers (cf. Fig. 1B), this proportion diminished to 6 %. Furthermore, under these conditions the bulk of the bound Azo-tagged POI (peak 2) eluted significantly earlier when triggering the elution by illumination with UV light at 355 nm. This is in agreement with the notion that under constant blue light (or daylight) exposure the mixture of ~25 % *cis*- and ~75 % *trans*-isomer behaves like a uniform molecular ensemble that exhibits binding activity towards α-CD (due to the presence of the Pap residues in the *trans*-configuration), however with overall lowered affinity. (B) Pictures of the column from (A) loaded with mScarlet-*Strep*-GG-Pap after washing with 0.6 or 2.0 mL chromatography buffer B, either in the dark or under 430 nm exposure. The *cis*-isomer (cAzo) and the *trans*-isomer (tAzo) become visibly separated during washing in the dark, whereas a homogeneous mixture is maintained under 430 nm exposure, which moves slightly faster

under buffer flow than the pure *trans*-isomer. (C) To investigate the influence of illumination with UV light for the protein elution from the α-CD affinity column, mScarlet-*Strep*-GG-Pap was applied to the column as in (A), followed by washing in the dark with 2 mL buffer. Elution was performed with 2 mL using (i) continuous exposure to 355 nm UV light (gray), (ii) exposure for only 3 min (with the remaining time in the dark, red), (iii) a short 1 min exposure (blue) or (iv) continuous but pulsed exposure (0.1 s on, 0.9 s off, 20 min in total; green). In case of the shortened exposure periods, elution was not fully quantitative (peak 2) since another 6-7 % of the Azo-tagged POI (peak 3) could be eluted from the column subsequently upon continued illumination with UV light. Pulsed 355 nm UV light led to slightly retarted, albeit complete elution. (D) α-CD affinity chromatography of the non-fluorescent blue-colored Azurin-*Strep*-GG-Pap, performed as in (A), here with chromatography buffer A, revealing similar effects. Azurin levels in each fraction were determined by measuring the absorbance at 620 nm with a Synergy 2 microtiter plate reader. Pictures of the column were taken after washing with 2 mL buffer. Numbering of peaks in all panels: 1, unbound *cis*-isomer that is immediately washed from the column under buffer flow; 2, fraction of the bound Azo-tagged POI that is eluted upon 355 nm UV light exposure; 3, fraction of the Azo-tagged POI which remained bound to the column when switching off the UV light before elution was complete.

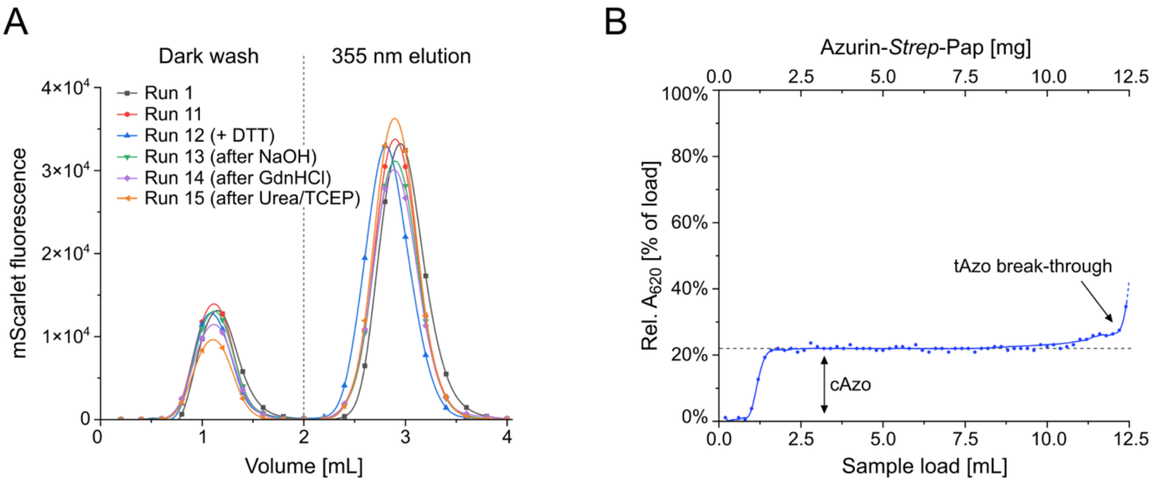

Suppl. Fig. S15. **Chemical stability and chromatographic performance of the α-CD affinity matrix.** (A) For chromatography Run 1 (gray), mScarlet-*Strep*-GG-Pap was loaded onto the affinity column (1 mL bed volume), followed by washing in the dark with chromatography buffer B, and elution was triggered by continuous UV illumination at 355 nm (for protein quantification, see Suppl. Fig. S14). Afterwards, the same column was used for affinity purification of different Azo-tagged POIs used throughout this study (Runs 2–10), including isolation from whole cell lysates, without noticeable change in performance, as seen for Run 11 (red), which was performed with mScarlet-*Strep*-GG-Pap again. Likewise, addition of 10 mM DTT to the sample and running buffer (Run 12, blue) or chromatography runs of mScarlet-*Strep*-GG-Pap performed after regeneration of the column for 1 h (Runs 13–15) with 1 M NaOH (green), 6 M guanidinium chloride (purple) or 8 M urea plus 10 mM TCEP (orange) did not affect the elution profile. (B) Determination of the dynamic column capacity. Purified Azurin-*Strep*-GG-Pap (1 mg/mL in chromatography buffer A) was loaded onto the α-CD affinity column (1 mL bed volume) and the characteristic absorption at 620 nm of this POI was recorded in 200 µL fractions. The initial shift in the baseline (dashed line) corresponds to the unbound proportion of the Azo-tagged POI with the Pap group in the *cis*-state (cf. Suppl. Fig. S14). After loading in total 11 mg of protein there was a tiny rise in the absorbance, which was followed by a steeper increase, corresponding to a 10 % breakthrough, after 12.2 mg of protein (0.8 µmol) had been applied. Thus, the molar dynamic capacity of the α-CD affinity column for an Azo-tagged 15.5 kDa protein corresponds to approximately 12 % of the capacity that was estimated for the free Pap amino acid (see Suppl. Methods).

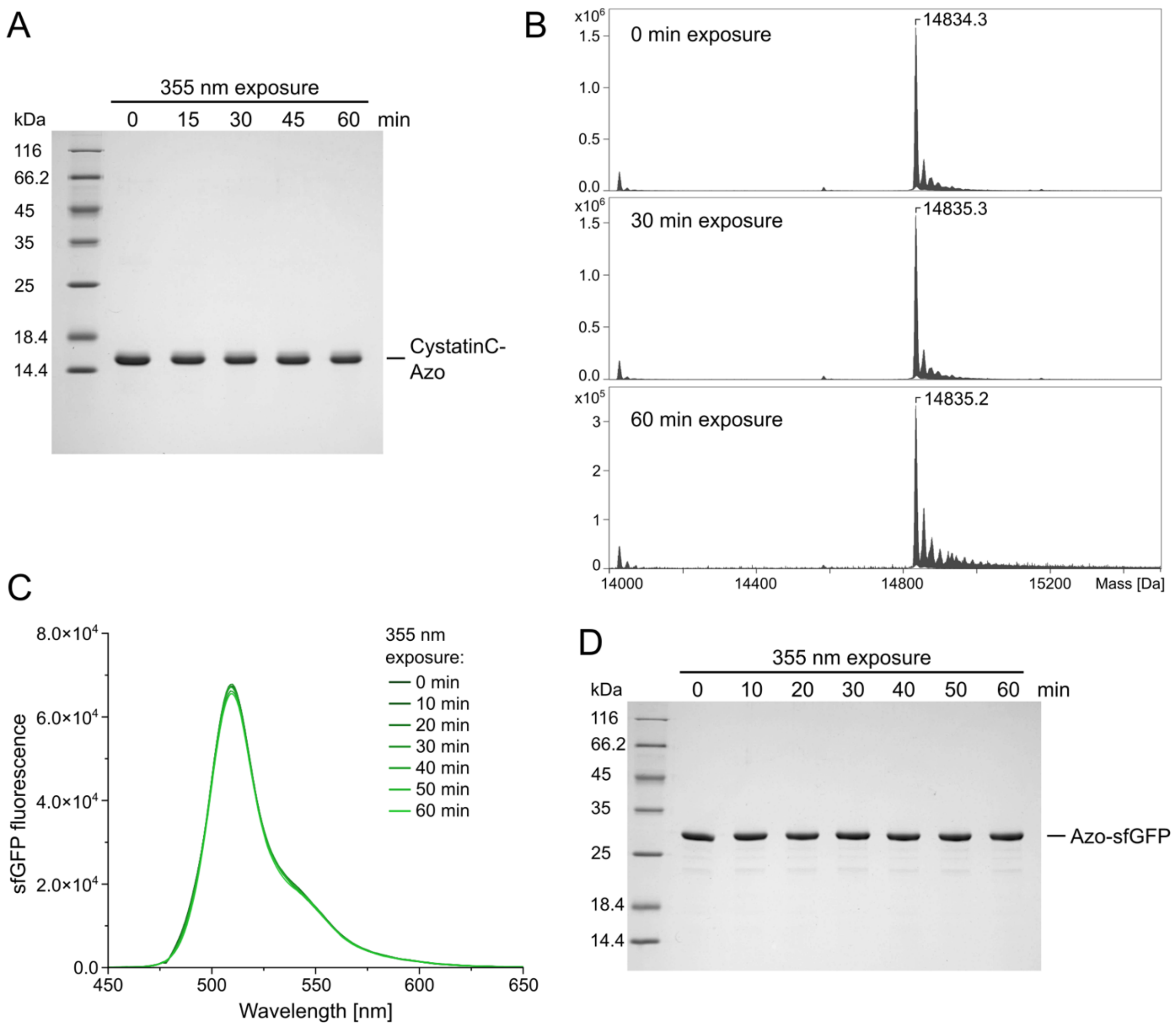

Suppl. Fig. S16. Influence of prolonged exposure to 355 nm UV light on the integrity of different POIs. (A) A 0.2 mg/mL solution of CystatinC-*Strep*-GG-Pap (CystatinC-Azo) in chromatography buffer A was applied to an empty column (without α-CD resin), which was placed into the setup otherwise used for the light-controlled affinity chromatography (cf. Suppl. Fig. 1), and exposed to 355 nm LED UV light for 0–60 min. Samples were collected every 15 min throughout the experiment and analyzed by SDS-PAGE without signs of degradation. (B) ESI-MS analysis of protein samples from (A) showing no effect of prolonged 355 nm exposure. (C) A 0.2 mg/mL solution of Gly-Pap-GP-sfGFP-*Strep* (Azo-sfGFP) in chromatography buffer A was exposed to 355 nm LED UV light for 0–60 min as in (A). No significant difference was observed in a superposition of the fluorescence spectra (λ_{Ex} = 488 nm) measured for all samples in a FluoroMax 3 spectrofluorimeter. (D) SDS-PAGE analysis of the Azo-sfGFP samples from (C), revealing a fully intact polypeptide chain.

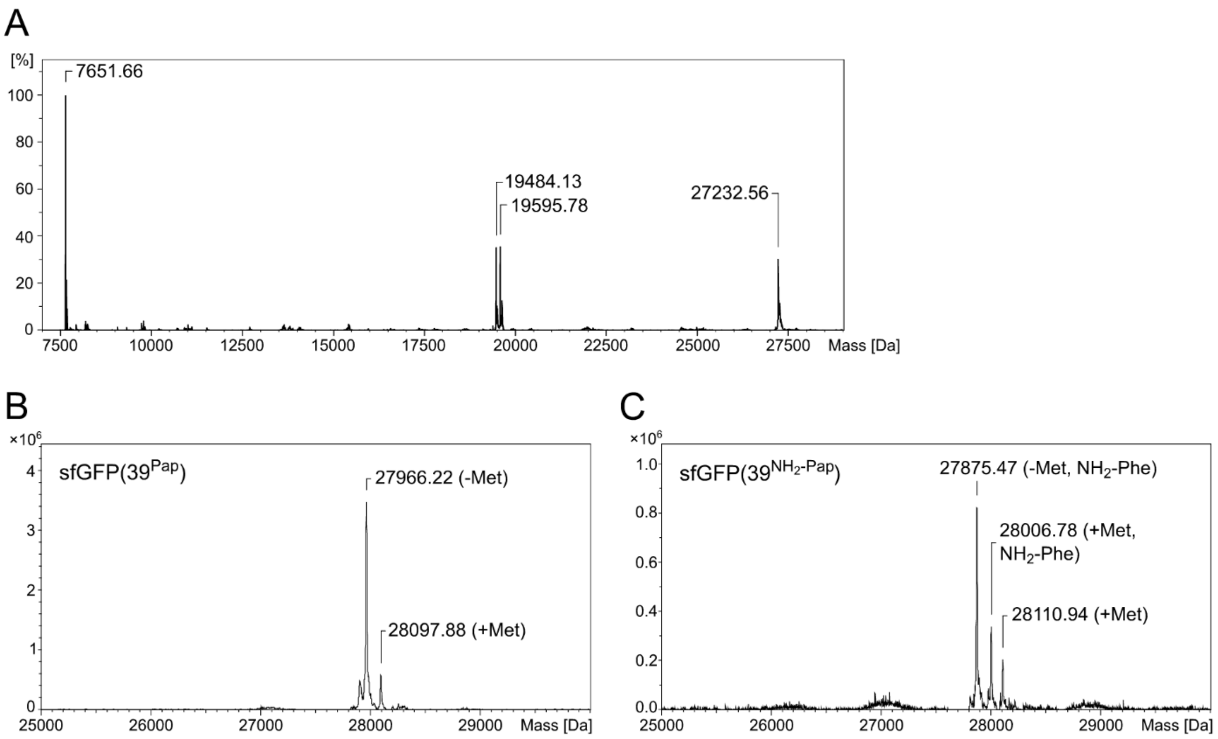

Suppl. Fig. S17. ESI-MS analyses of posttranslational modifications observed for POIs in this study. (A) Specific polypeptide bond cleavage in Gly-Pap-GG-mScarlet3. Apart from the intact mature fluorescent protein (calc. 27233.59 Da), this mass spectrum reveals a peak for an N-terminal fragment up to the residue preceding Met as part of the fluorophore (calc. 7653.56 Da) as well as for two distinct C-terminal fragments, one including the cyclized and oxidized fluorophore (calc. 19598.05 Da) and another one without cyclisation and missing the Met residue (calc. 19486.90 Da). (B, C) Reductive cleavage of NH_2-Pap after incorporation into sfGFPa39. Deconvoluted ESI mass spectrum of sfGFPa39 expressed in the presence of (B) Pap (calc. 27965.3 Da) or (C) NH_2-Pap (calc. 27980.5 Da). The shift of the major peak in the latter case indicates a possible reductive cleavage of the azo-bond, thus forming *p*-amino-L-phenylalanine (NH_2-Phe), which is not seen for Pap.

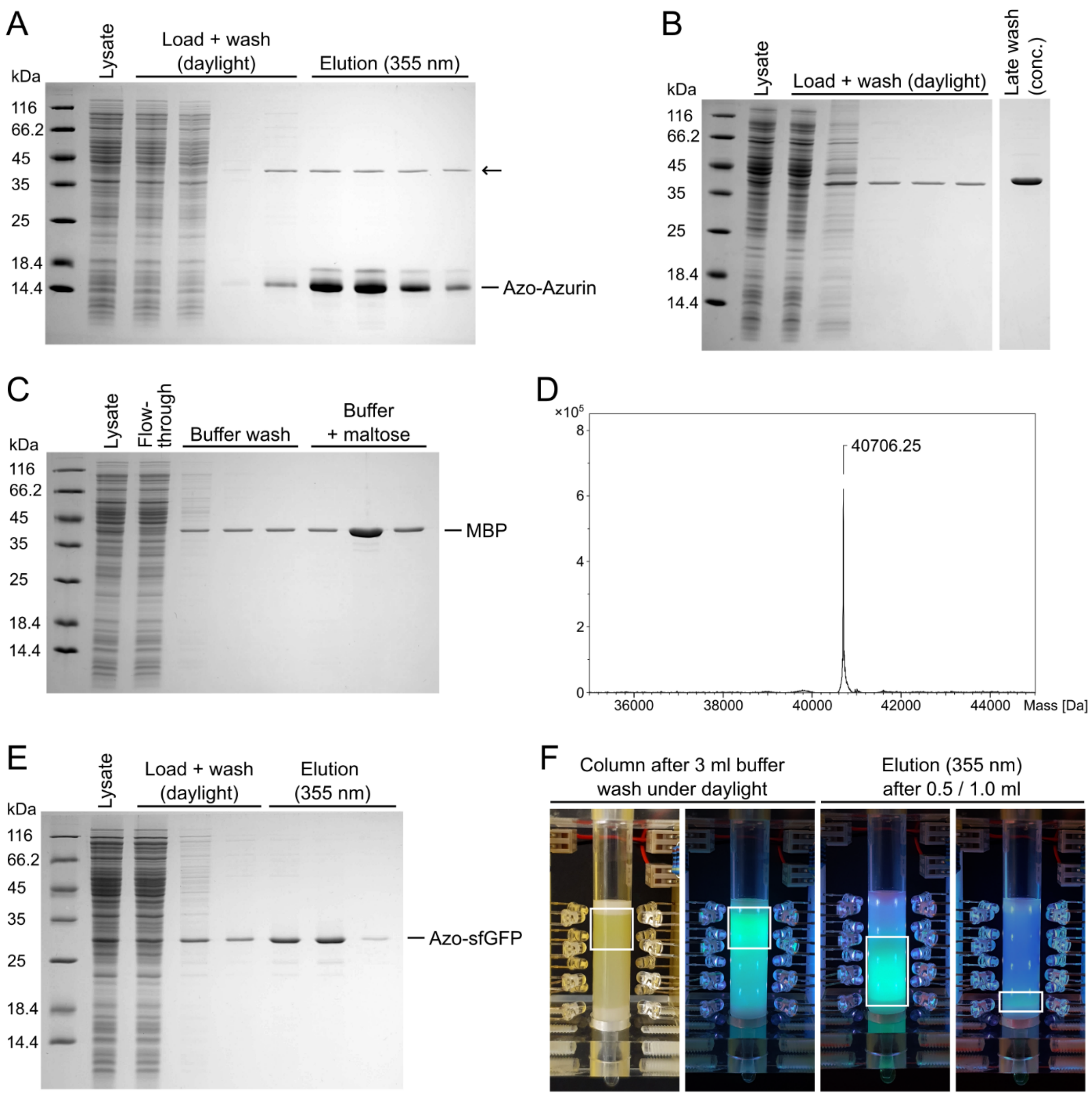

Suppl. Fig. S18. Identification of the bacterial MBP as a specific contaminant that reversibly adsorbs to the α-CD affinity matrix in a light-independent manner. (A) Purification of Ala-Pap-GG-Azurin-*Strep* from the total cell extract of NEBExpress(lowRF1) via α-CD affinity chromatography. Note the contaminating band (arrow), which slowly elutes irrespective of illumination and was later identified as MBP. (B) A total cell lysate of NEBExpress(lowRF1) was applied to the α-CD affinity column and all host cell proteins were washed out via buffer flow (chromatography buffer A) within 2 bed volumes, except for one protein that showed retarded elution and was still detectable after 10 bed volumes. (C) MBP from the bacterial lysate was specifically eluted when chromatography buffer A supplemented with 50 mM maltose was applied. (D) Late wash fractions were pooled, concentrated and the contaminating protein was identified as MBP (MalE) by ESI-MS analysis (calc. 40707.32 Da for the mature secreted protein). This protein was subjected to a trypsin digest and 16 peptide fragments were identified in the tandem MS/MS mode after RP-UHPLC, covering 58 % of the known amino acid sequence (UniProt ID: P0AEX9, excluding the signal peptide). (E) Purification of the intact sfGFP with N-terminal Gly-Pap-GP sequence via α-CD affinity chromatography from the total cell extract of NEBExpress(lowRF1/ΔMBP) in chromatography buffer A. No contamination by bacterial proteins was evident in the eluate, even though maltose was not added. (F) Photographs of the α-CD affinity column during purification of sfGFP as described in (E). sfGFP was retained at the top of the column after washing under daylight (left) but was efficiently eluted when exposed to 355 nm UV light (right).

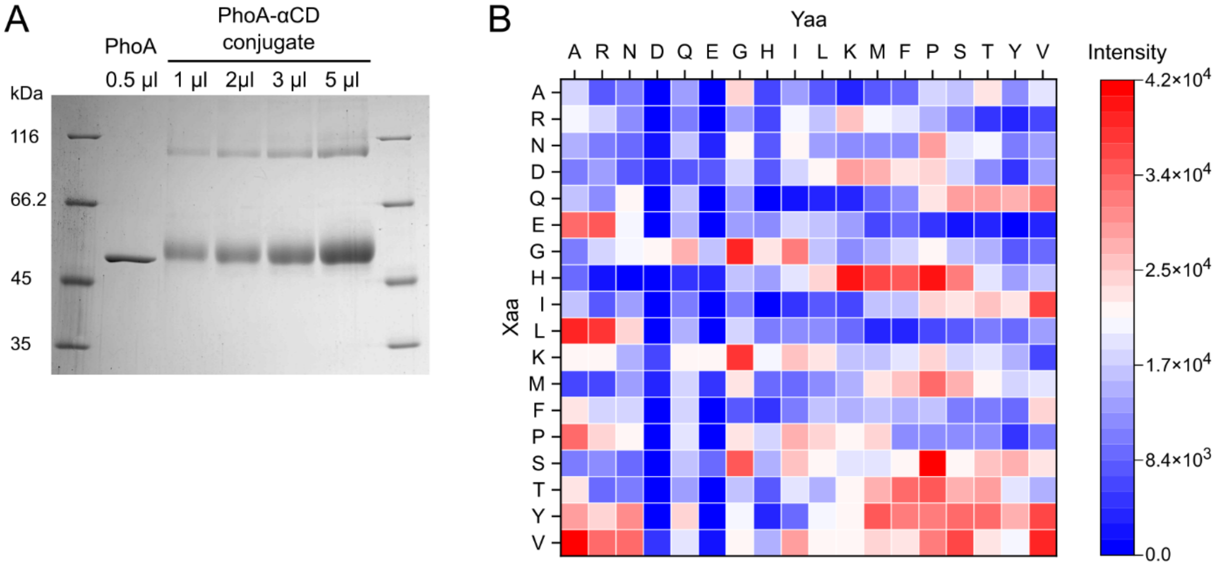

Suppl. Fig. S19. SPOT assay for optimizing the flanking amino acids on both sides of the Pap residue within the Azo-tag. (A) 15% SDS-PAGE of the α-CD-conjugated PhoA indicating an average labelling of 1 to 5 α-CD groups (~1100 Da) per enzyme subunit (47.2 kDa). The faint band at higher molecular weight presumably corresponds to the chemically cross-linked enzyme dimer. (B) Heat map representing the signal intensities after incubation of the SPOT membrane supporting C-terminally immobilized Xaa-Pap-Yaa peptides with the PhoA conjugate from (A), followed by chromogenic reaction. The identity of Xaa and Yaa residues in the synthetic peptide sequences is indicated on both axes.

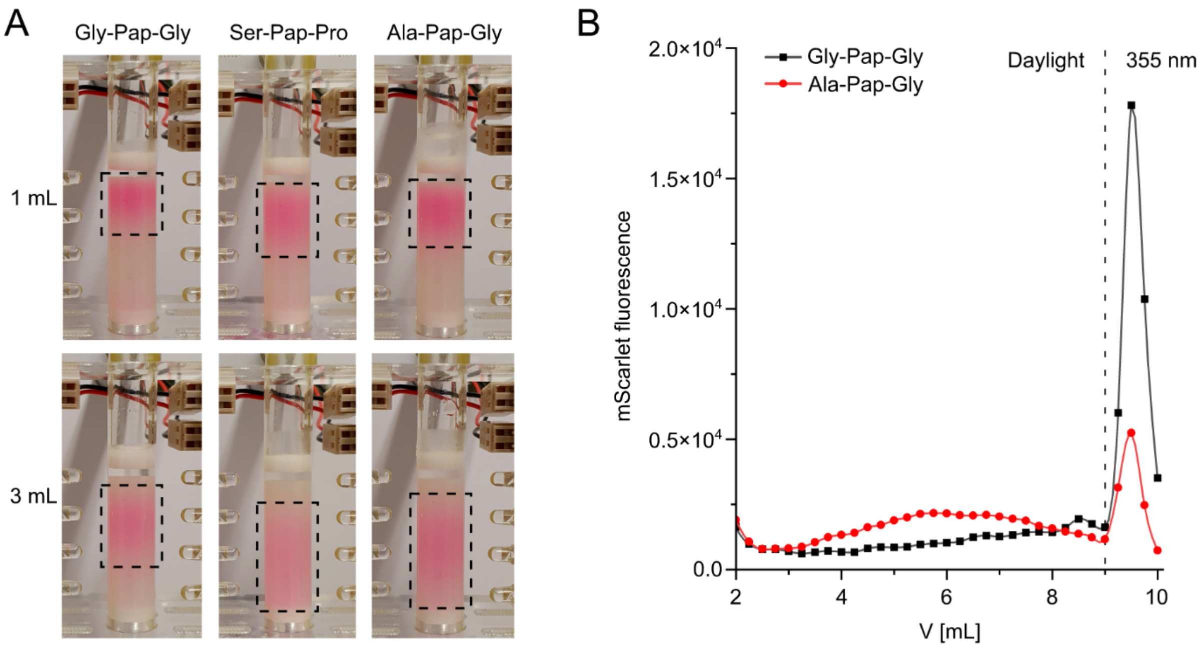

Suppl. Fig. S20. Comparison of different sequences surrounding the Pap residue in a recombinant protein carrying the N-terminal Azo-tag. (A) A total cell extract (1 mL) of NEBExpress(lowRF1) after expression of mScarlet3 equipped with an N-terminal Azo-tag comprising either Gly-Pap-Gly, Ser-Pap-Pro or Ala-Pap-Gly was loaded onto an α-CD affinity column (1 mL bed volume). This column is depicted after washing with 1 mL and (in total) 3 mL chromatography buffer A, respectively (without UV light exposure). The Gly-Pap-Gly version showed stronger retardation on the α-CD affinity matrix than Ala-Pap-Gly. The sequence Ser-Pap-Pro revealed inferior binding to the affinity column, despite its promising behavior in the SPOT assay (see Suppl. Fig. S19). (B) The α-CD affinity column was washed with a larger buffer volume than normally used (9 mL chromatography buffer A) prior to the UV light exposure, and mScarlet fluorescence was determined in 250 µl fractions using a Synergy 2 microtiter plate reader. Again, the Gly-Pap-Gly sequence led to lower protein loss during the washing steps, if compared with the Ala-Pap-Gly version, and higher yield of mScarlet3 eluted from the α-CD affinity column upon 355 nm UV light exposure.

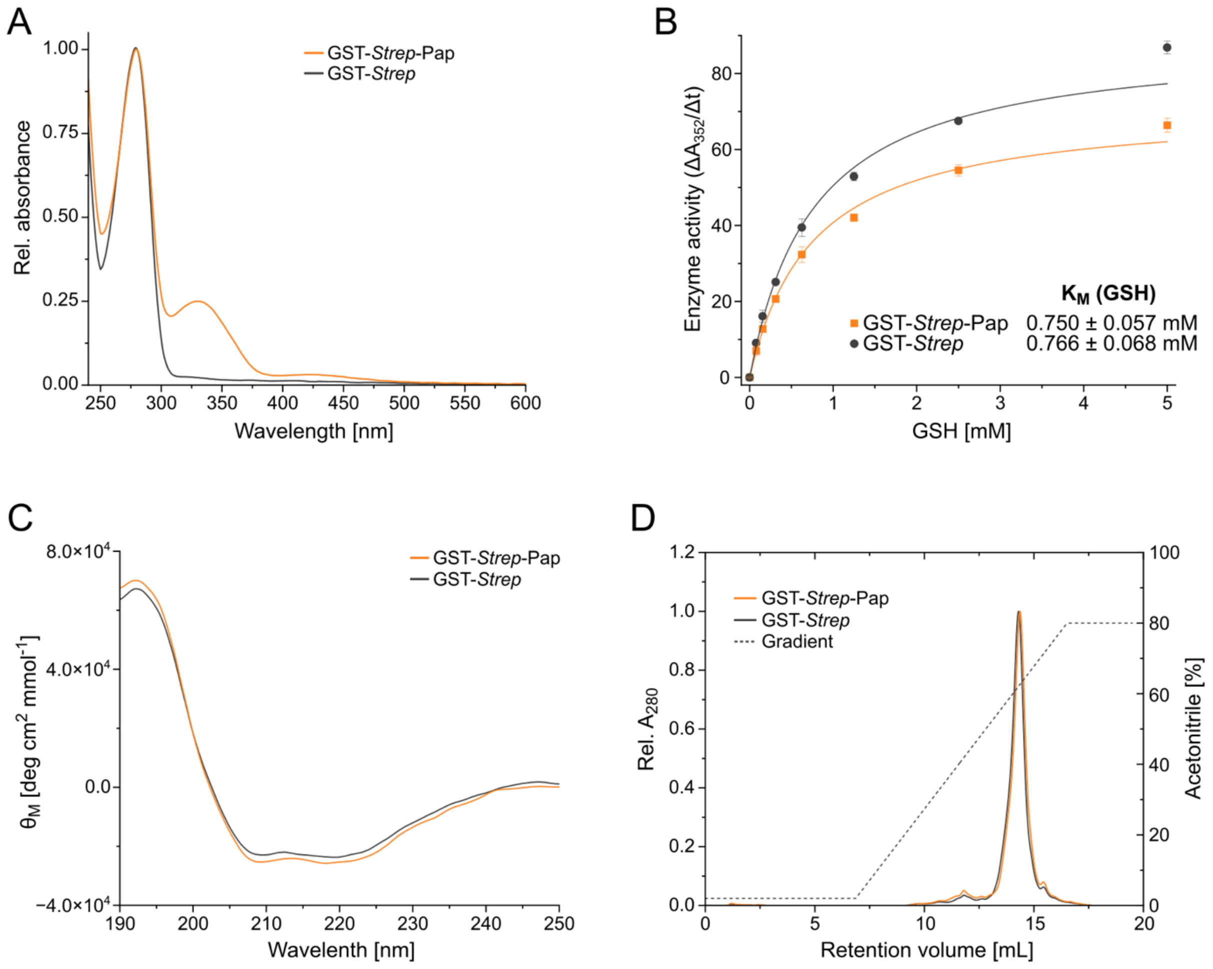

Suppl. Fig. S21. Potential influence of the Azo-tag on the biochemical properties and enzymatic function of GST as a typical POI. (A) Comparison of UV/Vis spectra of purified GST with and without a C-terminal Azo-tag (GST-*Strep*-GG-Pap *versus* GST-*Strep*-GG, respectively). While the characteristic absorption at 280 nm caused by the canonical Tyr and Trp side chains is present for both proteins, only the Azo-tagged GST reveals the specific absorption maximum around 330 nm that is contributed by the azobenzene side chain of Pap in its lower-energy *trans*-configuration. (B) Michaelis-Menten kinetic assay using 2.5 mM CDNB and varying concentrations of reduced L-glutathione (GSH) as substrates revealed K_M values identical within experimental error for both protein versions. The initial reaction velocity, v_0 , was measured with a Synergy 2 microtiter plate reader at 30 °C using GST assay buffer. (C) Circular dichroism (CD) spectra of both GST variants in GST assay buffer. The measured spectra were virtually identical and typical for proteins rich in α-helix, as is the case for GST, without any signs of changes in secondary structure or folding due to the presence of the Azo-tag. (D) Reversed phase chromatography was performed on a 1 mL prepacked Resource RPC column equilibrated in 2 % (v/v) acetonitrile, 0.1 % (v/v) formic acid. Both GST variants were eluted using a gradient of 2–80 % (v/v) acetonitrile, 0.1 % (v/v) formic acid. The two proteins essentially eluted at the same position in the chromatogram, corresponding to identical concentrations of the organic cosolvent, which indicates that the presence of the azobenzene side chain has no detectable influence on the overall surface hydrophobicity of the enzyme.

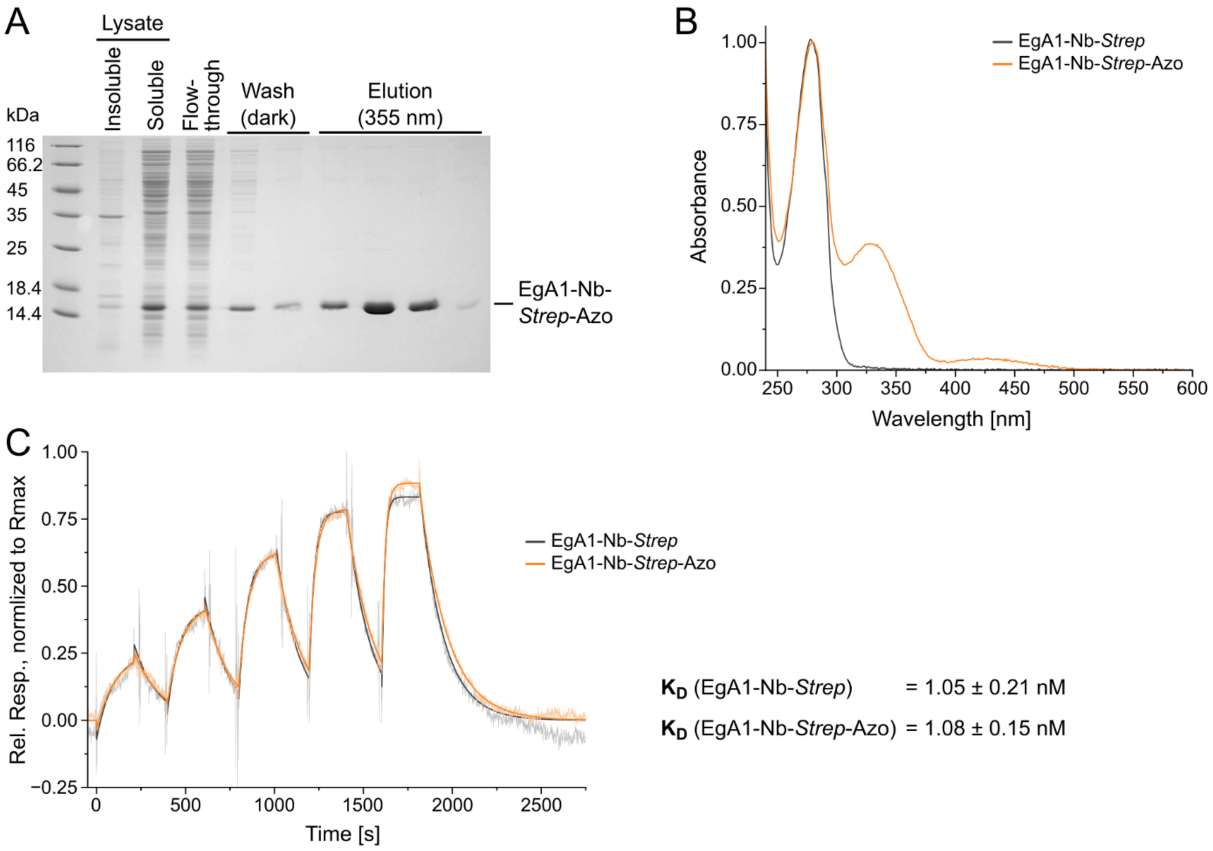

Suppl. Fig. S22. Purification via Excitography and functional analysis of an Azo-tagged nanobody (Nb), EgA1, directed against human epidermal growth factor receptor (hEGFR)[5]. (A) α-CD affinity chromatography of the αhEGFR Nb-*Strep*-GG-Pap, expressed in NEBExpress(lowRF1) using the pSB19-PapRS#34 vector, from the whole cell extract. (B) UV/Vis spectra of the purified αhEGFR Nb with and without a C-terminal Azo-tag (EgA1-Nb-*Strep*-GG-Pap *versus* EgA1-Nb-*Strep*-GG, respectively), revealing the specific absorption maximum around 330 nm of the Pap side chain (cf. Suppl. Fig. S21). (C) The antigen affinity of both αhEGFR Nb versions from (B) was measured by real-time surface plasmon resonance (SPR) on a Biacore X100 instrument. A hEGFR-Fc fusion protein was immobilized on a CM5 sensor chip via an amine-conjugated anti-Fc antibody, and "single-cycle" kinetic measurements were performed with a dilution series of the two Nb versions (light orange and light gray; raw data from exemplary measurements), followed by data fitting according to the 1:1 Langmuir model (orange and dark gray, respectively). The K_D values ± SD correspond to the mean and standard deviation of three individual measurements and confirm that the Azo-tag does not influence the antigen-binding function of the nanobody.

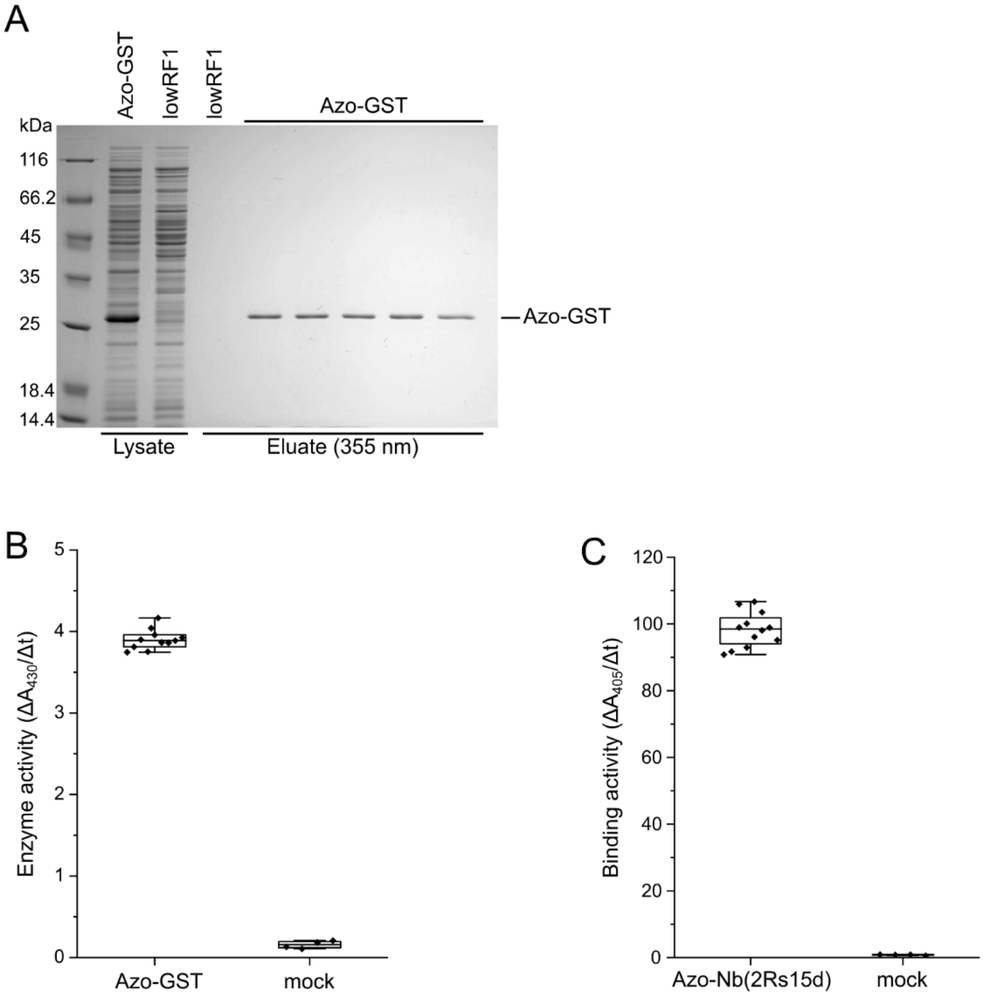

Suppl. Fig. S23. Light-controlled α-CD affinity chromatography of Azo-tagged GST and a nanobody in the microtiter plate format. (A) Isolation of GST carrying the N-terminal Gly-Pap-Gly sequence from the total cell extract of NEBExpress(lowRF1), applied (in 0.5 mL aliquots) to 11 wells of a 96-well receiver plate filled with 200 µl α-CD resin per well. Untransformed NEBExpress(lowRF1) served as a negative control (mock, four wells). Chromatography and UV light-induced elution were directly performed in the GST assay buffer. The SDS-PAGE shows the eluates of wells 1–5, demonstrating high protein purity and very low well-to-well difference. (B) GST enzyme activity was measured in the presence of GSH and CDNB (see Suppl. Fig. S21). (C) The αHER2 Nb 2Rs15d[6] was expressed with the N-terminal Gly-Pap-Gly sequence in the cytoplasm of NEBExpress(lowRF1), and the total cell extract (0.5 mL aliquots) was applied to 12 wells of the receiver plate as in (A). Antigen-binding activity in the eluted protein fractions was quantified on a HER2-coated microtiter plate via detection with an antibody-HRP conjugate directed against the *Strep*-tag II (StrepMAB-Classic HRP) using ABTS substrate. Box plots in (B) and (C) show all data points, each corresponding to a single well; median (center line), upper/lower quartiles (box limits), and min/max values (whiskers) are indicated.

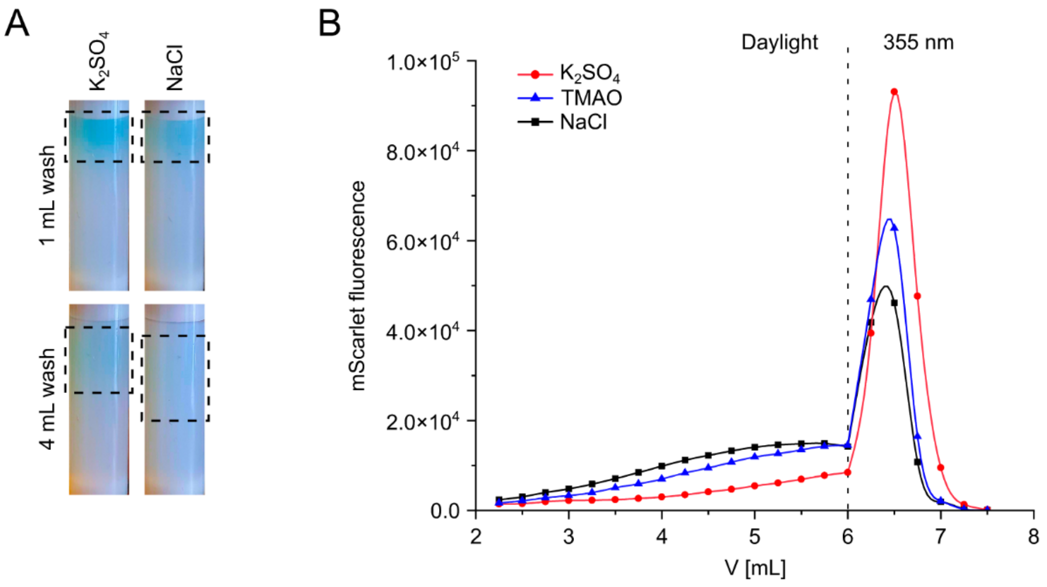

Suppl. Fig. S24. Influence of the buffer composition on the α-CD affinity chromatography. (A) Replacing NaCl by K_2SO_4 in the chromatography buffer (25 mM Tris/Cl pH 8.0, 150 mM NaCl *versus* 50 mM Tris/Cl pH 8.0, 100 mM K_2SO_4, dubbed chromatography buffer A and B, respectively) resulted in a slightly better retention of Azurin-*Strep*-GG-Pap on the α-CD column. (B) α-CD affinity chromatography was performed with mScarlet-*Strep*-GG-Pap using different buffer compositions, followed by quantification of the mScarlet fluorescence in 0.5 mL fractions with a Synergy 2 microtiter plate reader. The use of K_2SO_4 (with its higher ionic strength) throughout the chromatography resulted in some lower loss of protein during the extensive washing steps (6 mL in total) and higher yield upon the UV light-induced elution if compared to NaCl. Retention of mScarlet-*Strep*-GG-Pap was also increased when using the osmolyte trimethylamine N-oxide, TMAO (50 mM Tris/Cl pH 8.0, 0.5 M TMAO) instead of the salt component NaCl, albeit not to the same extent as with K_2SO_4.

A) C-terminal Azo-tag

Option 1: Azo-tag introduced with reverse primer; cloning via *Nde*I and *Hind*III restriction sites.

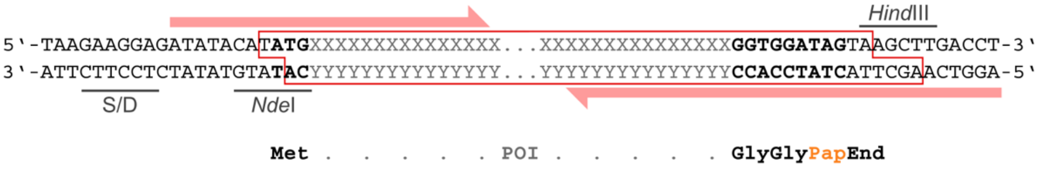

Option 2: pSB19 carries already a C-terminal tag preceded by an *Afe*I restriction site; cloning via sticky and blunt end ligation with *Nde*I/*Afe*I digested vector.

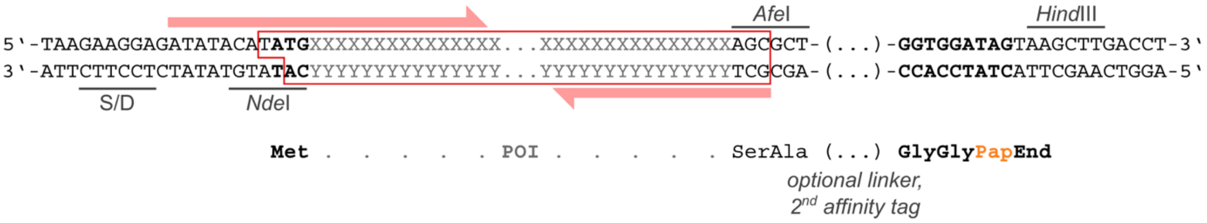

B) N-terminal Azo-tag (cytoplasmic expression)

Azo-tag introduced with forward primer; cloning via *Nde*I and *Hind*III restriction sites.

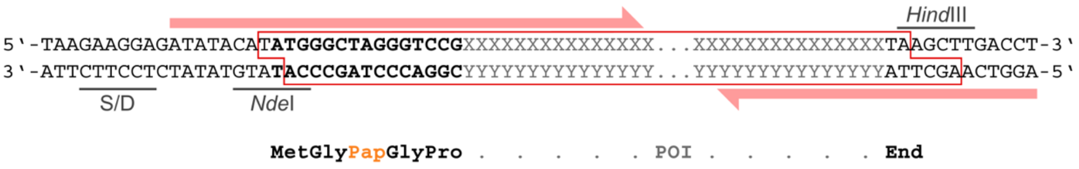

C) N-terminal Azo-tag (periplasmic secretion)

Azo-tag introduced with forward primer; cloning via blunt/sticky end ligation with a *Stu*I/*Hind*III digested pSB19-OmpA cloning vector.

pSB19-OmpA cloning vector:

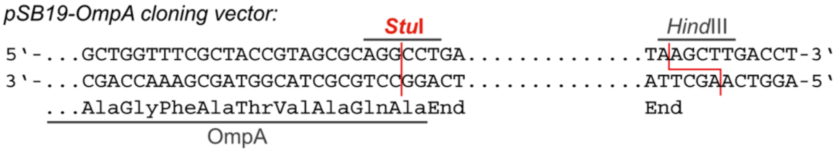

Cloning product:

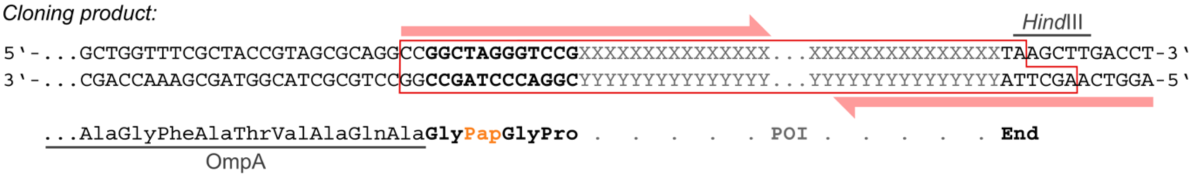

D) Cloning vector

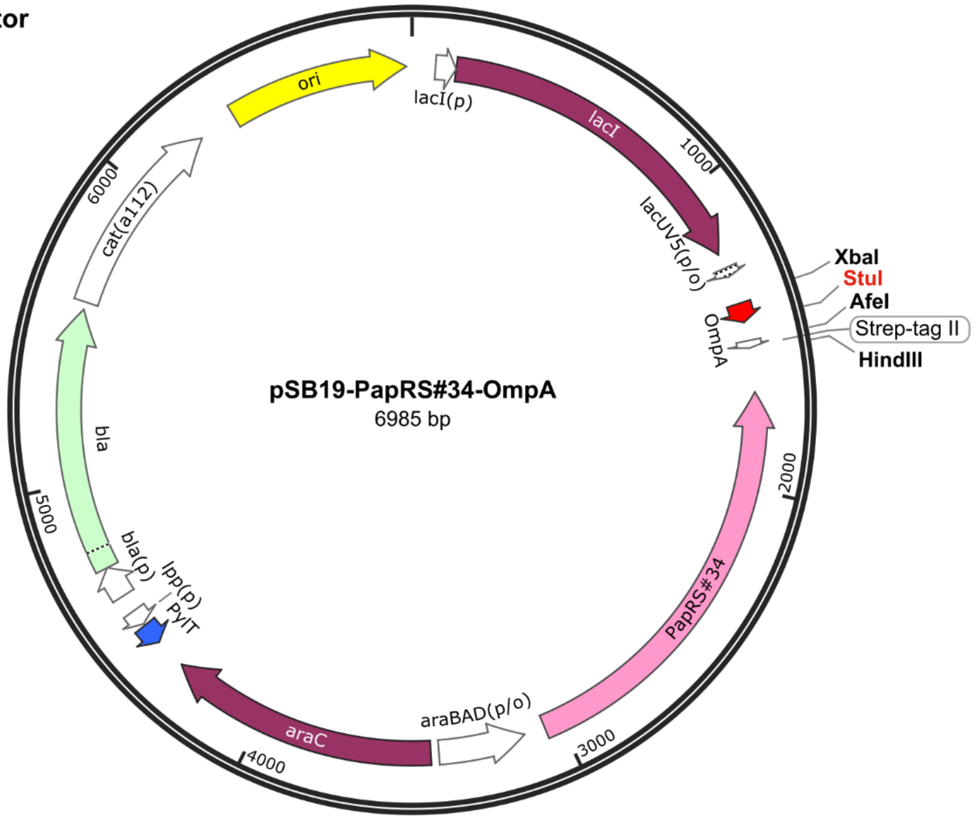

Suppl. Fig. S25. Sequence around the coding region on the pSB19-PapRS#34 expression vector (see Fig. 3A) and procedures to clone a gene for a new POI with the Azo-tag according to three different strategies. (A, B) Direct cytoplasmic expression with a C-terminal Azo-tag (A) or an N-terminal Azo-tag (B). Horizontal red arrows indicate the forward/backward PCR primers that are required to amplify the coding region for the POI and, concomitantly, create compatible ends with the pSB expression vector for one-step subcloning. Especially in the case of the C-terminal Azo-tag the use of a GGA triplet is recommended to encode the preceding Gly residue, which leads to more efficient suppression of the amber stop codon (TAG) that encodes the final Pap residue. Note that in the case of strategy (A) there is freedom of choice to additionally encode an N-terminal signal peptide for periplasmic secretion in *E. coli*. (C) Cloning strategy for a POI with an N-terminal Azo-tag utilizing the OmpA signal peptide already encoded on the cloning vector pSB19-PapRS#34-OmpA (D). On this vector the signal sequence is followed by an opal stop codon (TGA), which is eliminated when the new coding region of the POI is inserted via the *Stu*I restriction site.

Suppl. Table 1. List of Azo-tagged POIs used in this study. N-terminal tags correspond to the mature protein (i.e. with the OmpA signal sequence or fMet cleaved). C-terminal tags usually are preceded by a Ser-Ala sequence as encoded by an *Afe*I restriction site (AGCGCT), to facilitate subcloning.

Short name	POI	UniProt/PDB ID (aa residues)	N-terminal Azo-tag	C-terminal Azo-tag	Shown in Figures
**Azurin-Azo**	Azurin	1AZU (1–128)	–	Ser-Ala-*Strep*-tagII-Gly-Gly-Pap	4B, 4D, 5B, S12, S13A, S14D, S15B, S24A
**Azurin-GG-Pap**	Azurin	1AZU (1–128)	–	Ser-Ala-Gly-Gly-Pap	S13A
**Azo-Azurin**	Azurin	1AZU (2–128)	Ala-Pap-Gly-Gly	Ser-Ala-*Strep*-tagII	S18A
**mScarlet-Azo**	mScarlet	XCO01338 (1–232)	–	Ser-Ala-*Strep*-tagII-Gly-Gly-Pap	4B–C, 5C, S13B, S14A–C, S15A, S24
**Azo-mScarlet3**	mScarlet3	7ZCT (2–229)	Ala-Pap-Gly-Gly	Ser-Ala-*Strep*-tagII	5D, S20
**G-Pap-G-mScarlet3**	mScarlet3	7ZCT (2–229)	Gly-Pap-Gly-Gly	Ser-Ala-*Strep*-tagII	S7A, S20
**S-Pap-P-mScarlet3**	mScarlet3	7ZCT (2–229)	Ser-Pap-Pro-Gly	Ser-Ala-*Strep*-tagII	S20A
**Azo-sfGFP**	sfGFP	2B3P (2-237, K238Q)	Gly-Pap-Gly-Pro	Ser-Ala-*Strep*-tagII	S16C–D, S18E–F
**Cystatin-Azo**	Human cystatin C	1G96 (1–119)	–	Ser-Ala-*Strep*-tagII-Gly-Gly-Pap	5A, S16A–B
**CytB5-Azo**	Rat cytochrome B5	1I87 (1-98)	–	Gly-Gly-Pap	–
**Azo-ProtG**	Protein G C2 domain	1FCC (1–56)	Gly-Pap-Gly-Pro	Ser-Ala-*Strep*-tagII	6D
**AmpC-Azo**	AmpC	ACN91302 (1–361)	–	Ser-Ala-*Strep*-tagII-Gly-Gly-Pap	6A–B
**Azo-GST**	GST	1GNE (1-217; C84S/C137S/C177S)	Gly-Pap-Gly-Gly	Ser-Ala-*Strep*-tagII	S23A–B
**GST-*Strep*-Azo**	GST	1GNE (1-217; C84S/C137S/C177S)	–	Ser-Ala-*Strep*-tagII-Gly-Gly-Pap	S21
**scFv(T84.66)-Azo**	αCEA scFv	1MOE (humanized)	–	Ser-Ala-*Strep*-tagII-Gly-Gly-Pap	6C
**Azo-Nb(2Rs15d)**	αHER2 Nanobody	5MY6 (1-114)	Gly-Pap-Gly-Gly	Ser-Ala-*Strep*-tagII	S23C
**Nb(EgA1)-*Strep*-Azo**	αEGFR Nanobody	4KRN (1-129)	–	Ser-Ala-*Strep*-tagII-Gly-Gly-Pap	S22

Supplementary Methods

Chemicals and substrates

4-Amino-(Fmoc-phenylalanine) was purchased from Biosynth (Staad, Switzerland), nitrosobenzene and α-CD were obtained from Merck Millipore (Darmstadt, Germany). For experiments with β-CD, the derivative (2-hydroxypropyl)-β-cyclodextrin (Merck Millipore) was used throughout due to its superior solubility. Mono-2-O-(p-toluenesulfonate) α-CD was from TCI (Tokio, Japan). GSH was purchased from AppliChem (Darmstadt, Germany), NADPH and tris(2-carboxyethyl)phosphine (TCEP) were from Carl Roth (Karlsruhe, Germany) and TMAO was from Acros Organics (Geel, Belgium). GSH and CDNB were purchased from Sigma-Aldrich (Taufkirchen, Germany), CENTA was from Merck Millipore and ABTS was from Roche Diagnostics (Mannheim, Germany). Phenylalanine-4'-azobenzene (Pap) HCl was procured as reference substance from Sigma-Aldrich / Merck.

Synthesis of p-(phenylazo)-L-phenylalanine (Pap; Suppl. Fig. S2A)

Nitrosobenzene (6.70 g, 62.6 mmol) and 4-amino-(Fmoc-L-phenylalanine) (25 g, 62.2 mmol) were dissolved in 200 mL dimethylsulfoxide (DMSO), resulting in a clear green solution. Then, 200 mL acetic acid was added, followed by stirring at room temperature for 24 h. The resulting suspension was poured into 1.5 L ice-cold water and stirred for 10 min. The beige precipitate was filtered off and washed twice with 200 mL cold water. The damp solid (Fmoc-Pap-OH) was air-dried for 72 h. The residue was dissolved in 200 mL dimethylformamide (DMF), to which 50 mL piperidine was added, and stirred for 3 h at room temperature. 750 mL cold water was then added, and the pH was brought to approximately 6.0 with conc. HCl followed by stirring for 2 h, which resulted in a turbid suspension. The suspension was extracted 3 times with 200 mL diethyl ether; the organic phase was discarded, and the precipitate (Pap in the zwitterionic state) was separated by filtration and washed twice with water and diethyl ether. After air drying, 16.1 g of orange powder was obtained (60.0 mmol; 96 %).

^{1}H-NMR (400 MHz, DMSO-d_6/D_2O/NaOD): δ = 7.79 (d, J = 7.3 Hz, 2H, CH), 7.74 (d, J = 8.0 Hz, 2H, CH), 7.54-7.48 (m, 3H, CH), 7.39 (d, J = 8.0 Hz, 2H, CH), 3.30-3.28 (m, 1H, αCH), 3.01-2.98 (m, 1H, CH_2) 2.70-2.67 (m, 1H, CH_2) ppm. ^{13}C-NMR (100 MHz, DMSO-d_6/D_2O/NaOD): δ = 180.9, 153.1, 151.6, 144.9, 132.8, 131.7, 130.8, 123.7, 123.6, 58.3, 42.2 ppm. ESI-MS(-): $[M–H^+]^-$ = 268.12 Da (calc. 268.11 Da)

To prepare the Na-salt, 200 mg Pap from above was dissolved in 30 mL 1 M HCl und loaded onto a cation exchange resin (Fractogel EMD SO_3^-; Merck Millipore) equilibrated with 1 M HCl. After washing with 500 mL 1 M HCl a bright orange fraction was eluted with 0.1 M NaOH. The fractions were collected and freeze-dried over night, yielding an orange solid.

Preparation of N-Fmoc-p-(phenylazo)-L-phenylalanine (Fmoc-Pap-OH)

To obtain analytically clean Fmoc-Pap-OH, the air-dried crude intermediate from the Pap synthesis described above was recrystallized from toluene as follows: the crude brown product (3.00 g) was refluxed in toluene (~1 L) for 15 min and hot filtered through a ceramic frit. The filtrate was cooled to -20°C overnight to yield a microcrystalline orange solid, which was collected on a filter and washed twice with pentane (2.21 g).

Synthesis of 4-(4-aminophenylazo)-L-phenylalanine dihydrochloride (NH_2-Pap; Suppl. Fig. S2B)

Step 1: Synthesis of 4-nitroso-acetanilide

4-Amino-acetanilide (10.0 g, 66.0 mmol) was dissolved in 1.0 L water, resulting in a clear brownish solution, which was placed into an ice bath and equilibrated under stirring for 15 min. Oxone (81.2 g, 132 mmol) was dissolved in 500 mL water and carefully basified with

solid K_2CO_3 (25.0 g; 181 mmol). The Oxone solution was slowly added (~30 min) to the ice-cooled 4-amino-acetanilide solution, which resulted in the formation of a green precipitate. The reaction was completed by stirring at room temperature for 15 min. The green solid was recovered by filtration and washed twice with 50 mL water. After drying on air overnight the yield of the olive-green residue was 10.0 g (60.6 mmol; 92 %).

Step 2: Synthesis of 3-(4-((4-acetamidophenyl)diazenyl)phenyl)-2-aminopropanoic acid
4-Nitroso-acetanilide (10.0 g, 60.6 mmol) from above and 4-amino-(Fmoc-L-phenylalanine) were dissolved in 250 mL DMSO, resulting in a clear brown solution. 250 mL acetic acid was added and the solution was stirred overnight. The solution was poured into 2.5 L ice-cold water and stirred for 10 min. A beige precipitate formed, which was recovered by centrifugation and washed twice with water. The solid was resuspended in 200 mL water and 100 mL piperidine was added. After stirring for 2 h at room temperature, the mixture was extracted with 200 mL diethyl ether 3 times to remove the 9-methylene fluorene that had formed during the deprotection step, and the combined organic phases were discarded. The solid residue was recovered from the aqueous phase by filtration. The crude product was mainly composed of 3-(4-((4-acetamidophenyl)diazenyl)phenyl)-2-aminopropanoic acid and directly used for the next synthesis step.
ESI-MS(-): $[M–H^+]^-$ = 325.13 Da (calc. 325.13 Da)

Step 3: Synthesis of 4-(4-aminophenylazo)-L-phenylalanine dihydrochloride
The wet crude product from step 2 was resuspended in 100 mL water. Then, 200 mL conc. HCl was added, upon which the brown suspension turned dark red. The mixture was heated under reflux for 3 h until a clear blood-red solution was obtained. The hot solution was evaporated at 60°C in a rotary evaporator at 1 mbar, and the red residue was extracted twice with 250 mL acetone. The red solid was recovered by filtration and washed with 100 mL acetone, then dried under vacuum, yielding 6.50 g (18.2 mmol; 30 %) of a dark red powder.
^{1}H-NMR (400 MHz, DMSO-d_6): δ = 8.65 (bp, NH_3), 7.82 (d, J = 8.0 Hz, 2H, CH), 7.78 (d, J = 8.0 Hz, 2H, CH), 7.48 (t, J = 8.0 Hz, 2H, CH), 7.12 (t, J = 8.0 Hz, 2H, CH), 4.21-4.15 (m, 1H, αCH), 3.29-3.21 (m, 2H, CH_2) ppm. ^{13}C-NMR (100 MHz, DMSO-d_6): δ = 170.6, 151.5, 146.7 (bp), 145.6 (bp), 138.1, 131.1, 125.3, 122.6, 118.9, 53.5, 35.8 ppm. ESI-MS(+): $[M+H^+]^+$ = 285.14 Da (calc. 285.13 Da)

Preparation of α-CD Sepharose

3.1 g Epoxy-activated Sepharose 6B (Cytiva, Freiburg, Germany) was soaked and washed 5 times with 20 mL water in a 25 mL glass reaction vessel equipped with a frit. 1.0 g α-CD was dissolved in 10 mL 10 mM NaOH and added to the slurry, followed by gentle shaking at 37 °C for 18 h. The slurry was washed three times with 20 mL water, then 10 mL 1 M ethanolamine/HCl pH 8.0 was added and the mixture was agitated at 37 °C for further 4 h to block remaining reactive groups. Finally, the functionalized Sepharose was drained and washed five times with 20 mL water, then three times with 25 mM Tris/HCl pH 8.0, and kept in this buffer for storage.

Quantification of the immobilized α-CD groups on the Sepharose matrix

45 mg α-CD-conjugated Sepharose from above, or epoxy-activated Sepharose blocked with ethanolamine, both dried *in vacuo* for 48 h over P_2O_5, were placed in 1.5 mL reaction tubes. After soaking with 0.5 mL water for 15 min and vortexing for 1 min, the wet residue was separated by centrifugation (2 min) and the supernatant was discarded. The matrix was mixed with 5 mM Pap in 0.1 M NaOH by vortexing for 30 s and incubated for 5 min. After

centrifugation and removal of the supernatant, the residue was washed twice with 0.5 mL 0.1 M NaOH (30 s vortex, 1 min incubation, 2 min centrifugation). After these washing steps a distinct difference in color was visible: while the reference matrix was almost colorless, the α-CD-conjugated Sepharose remained intensely orange (cf. Suppl. Fig. S9). Both residues were resuspended in 1.0 mL 0.1 M NaOH and transferred each into a Pierce disposable 2 mL column, followed by irradiation with 355 nm UV light for 10 min. The columns were drained and both eluates collected in 2 mL disposable polystyrene cuvettes (Sarstedt, Nümbrecht, Germany). The almost colorless eluate from the reference matrix was used as baseline and the difference in absorption at 426 nm was immediately measured. Using the experimentally determined extinction coefficient for *cis*-Pap (see text) a capacity of 6.5 μmol/mL wet volume Sepharose was estimated.

<u>Light-dependent binding of Pap to α-CD sepharose</u>

1 mL of α-CD-conjugated Sepharose resuspended in 25 mM Tris/HCl pH 8.0 was packed into a Pierce disposable 2 mL column. Then, 100 μl of a 5 mM Pap solution in 10 mM NaOH was applied and allowed to soak into the column under gravity flow. When the column was rinsed with 1 mL 100 mM Tris/HCl pH 8.0, 500 mM NaCl, Pap was retained at the top of the column as indicated by its pronounced yellow color (Suppl. Fig. S9). The same procedure was followed for a negative control using a Sepharose reference (epoxy-activated Sepharose blocked with ethanolamine). In this case, Pap immediately eluted with the buffer flow (1.0 mL). When the first column was further rinsed with 10 mL 100 mM Tris/HCl pH 8.0, 500 mM NaCl, the yellow zone showed only minor movement. However, after 10 min lateral illumination with 355 nm UV light, the yellow band immediately eluted when applying 1.5 mL of the same buffer. Immediate measurement of a UV/Vis spectrum of the eluate revealed the characteristic absorption pattern of pure *cis*-Pap (cf. Fig. 2D and Suppl. Fig. S9B).

<u>Preparation of an α-CD-conjugated enzyme</u>

Bacterial alkaline phosphatase (PhoA) carrying the C-terminal *Strep*-tag (II) was produced in *E. coli* and purified to homogeneity from the periplasmic cell extract via StrepTactin affinity chromatography as previously described[7], yielding a 1.5 mg/mL solution in PBS (7 mL). α-CD mono-2-O-(p-toluenesulfonate) (100 mg, 89 μmol) was dissolved in 1 mL 25 % (w/v) aqueous NH_3 under stirring for 7 d to complete the aminolysis (as checked by ESI-MS). The resulting α-CD mono-amine was precipitated with acetonitrile and washed twice. After drying *in vacuo*, 85 mg of a white powder was obtained (87 μmol; 98 %).
ESI-MS(+): $[M+H^+]^+$ = 972.34 Da (calc. 972.34 Da).
Next, the α-CD mono-amine (35 mg, 36 μmol) was dissolved in 200 μL dry DMF and glutaric anhydride (5 mg, 440 μmol) was added, followed by stirring for 30 min at room temperature. The reaction product, α-CD mono-2-amino-glutarate, was precipitated with acetonitrile and washed twice with the same solvent. After drying *in vacuo*, 25 mg of a white powder was obtained (23 μmol; 64 %).
ESI-MS(-): $[M{-}H^+]^-$ = 1084.39 Da (calc. 1084.36 Da).
The α-CD mono-2-amino-glutarate (10 mg, 9.2 μmol) as well as N-hydroxy-succinimide (NHS; 3.0 mg, 26 μmol) and 1-ethyl-3-(3-dimethylaminopropyl)carbodiimide hydrochloride (EDC; 2.0 mg, 10 μmol) were dissolved in 200 μL dry DMF and incubated for 1 h at room temperature. ESI-MS analysis of this solution confirmed the formation of essentially clean α-CD mono-2-amino-glutaryl NHS-ester.
ESI-MS(+): $[M+H^+]^+$ = 1183.41 Da (calc. 1183.39 Da).

50 μL of this NHS-ester solution in DMF was gently mixed with 2 mL of the purified PhoA from above (1.5 mg/mL in PBS, adjusted to pH 8.5 with 0.1 M NaOH). After incubation for 2 h, the conjugated protein was separated from the reagents using a PD-10 column equilibrated with PBS. The α-CD-PhoA fractions were collected and combined, yielding 3 mL with a concentration of 0.7 mg/mL. SDS-PAGE analysis showed successful conjugation of the enzyme with α-CD groups as evident from the increase in size (Suppl. Fig. S19A).

SPOT synthesis of an immobilized peptide array including Pap residues

An array of trimer peptides, Xaa-Pap-Yaa, with Xaa and Yaa representing all amino acids except for the bulky Trp and the chemically reactive Cys (324 peptides in total) was synthesized with an automated MultiPep RS instrument (Intavis, Cologne, Germany) according to the SPOT method[8]. Solid phase peptide synthesis was performed on a Gly-PEG500-derivatised cellulose-membrane (Intavis), starting from the C-terminus and using Fmoc-protected amino acids. To keep the N-terminus unmodified (as it would happen at the N-terminus of a recombinant protein), a final capping step with acetic anhydride was omitted. After deprotection of the peptides, the membrane was rehydrated and blocked overnight at 4 °C with 10 % (w/v) blocking reagent (Roche Diagnostics) in membrane blocking solution (MBS; 50 mM Tris/HCl pH 8.0, 137 mM NaCl, 2.7 mM KCl, 0.05 % (v/v) Tween-20, 1 % (w/v) sucrose). After washing for 10 min in TBS/T (50 mM Tris/HCl pH 8.0, 137 mM NaCl, 2.7 mM KCl, 0.05 % (v/v) Tween-20) the membrane was incubated for 1 h at room temperature with a 1 µM solution of the α-CD-PhoA conjugate (see above) diluted in TBS/T. Then, the membrane was washed twice each with TBS/T and TBS (without Tween-20) for 5 min, followed by a short washing step with AP buffer (100 mM Tris/HCl pH 8.8, 100 mM NaCl, 5 mM $MgCl_2$). Finally, the signals were developed in 20 mL AP buffer with the addition of 60 µL 5-bromo-4-chloro-3-indolylphosphate p-toluidine salt (BCIP; 50 mg/mL in DMF) and 10 µL 2,2'-bis(4-nitrophenyl)-5,5'-diphenyl-3,3'-(3,3'-dimethoxy-4,4'-diphenylene)ditetrazolium chloride (NBT; 75 mg/mL in 70 % (v/v) DMF) for approximately 5 min. After washing the membrane extensively with water, the color signals were digitized with a Perfection V700 Photo scanner (Epson, Düsseldorf, Germany) and the spot intensities were quantified (in arbitraty units, a.u.) using Quant version 12.2 software (TotalLab, Newcastle-Upon-Tyne, UK). The resulting numbers were converted into a heat map using Excel software (Microsoft, Munich, Germany) (Suppl. Fig. S19B).

Supplementary Results

Quantification of the wavelength-dependent photostationary composition of Pap *cis*- and *trans*-isomers using ^{1}H-NMR spectroscopy and measurement of the relaxation half-life of the *cis*-state

Pap (Na-salt; 2 mg) was dissolved in 1.5 mL D_2O and 5 µL DMSO was added (serving as internal NMR standard with a final concentration of 50 mM) to yield a clear yellow solution (~4.5 mM Pap). For triplicate measurements, three NMR tubes (Sigma-Aldrich ColorSpec, 5 mm × 7 in.) were each filled with 500 mL of this solution and initially equilibrated under daylight (DL) for 2 h (avoiding both direct sunlight and artificial lighting). ^{1}H-NMR spectra were collected at 298 K on an Avance Neo 500 MHz instrument (Bruker, Rheinstetten, Germany) equipped with a cryoprobe (CP 2.1 TCI 500 S2 H–C/N-D-05 Z XT).

For irradiation experiments, the solution in each tube was illuminated (in a shaded, almost dark room) for 5 min with a row of four LEDs, placed at a short distance along the NMR tube, emitting peak wavelengths of 355, 365, 400, 410, 420, 430, 435, 440 or 450 nm. After that, the tube was immediately applied to the NMR instrument and the spectrum was measured (8 scans). For thermal relaxation experiments under acidic conditions, 30 µL of conc. HCl was added to 1.5 mL of sample as above and the triplicates were incubated in the dark for 24 h at 25°C prior to the NMR measurement.

Data acquisition was performed using TopSpin 4.1.1 software (Bruker). *trans*:*cis* isomer ratios were determined with MestReNova 15.0.1 software (Mestrelab Research, La Coruña, Spain) using the sum of the peak integrals of the aromatic protons (see Suppl. Fig. S4 and S5) relative to the singulet peak of the DMSO standard.

For kinetic stability experiments with *cis*-Pap, a 4 mL (1x1 cm^2) quartz cuvette was filled with 1980 µL 100 mM Tris/HCl pH 8.0. 20 µL of a 5 mM Pap solution in 10 mM NaOH was added and thoroughly mixed by pipetting. The sample was irradiated with UV light at 355 nm for 60 min at 25 °C as described above (see Suppl. Fig. S1). Immediately after transfer into a 1 cm pathlength quartz cuvette sealed with a Teflon screwcap (STARNA, Pfungstadt, Germany), the absorbance at 326 nm was quantified via brief measurement (in total <1 s) every 12 h over a period up to 96 h (Fig. 2F). Absorbance values were corrected by the blank spectrum of the buffer in the same cuvette.

The first order decay was fitted to the equation $A(t) = A_0 + A\cdot(1-e^{-k\cdot t})$, with half-life $t_{1/2} = \ln2/k$ and knowledge of the amplitude $A = 0.958 \pm 0.014$ (and also $A_0 = 0.2006 \pm 0.0051$, at the start of the experiment) from the preparation of the *cis*-state via illumination with UV light at 355 nm (as shown in Fig. 2E).

Directed evolution of aPylRS to efficiently charge its cognate amber suppressor tRNA with Pap or NH_2-Pap

An amino-acyl tRNA synthetase derived from *M. mazei* PylRS, which had been selected to incorporate *meta*-substituted derivatives of Pap for use in click chemistry, dubbed MmPSCaaRS[9], was used as a starting point to develop an enzyme that can efficiently charge $tRNA^{Pyl}$ with the amino acid Pap, carrying a plain azobenzene side chain, or its *para*-substituted, more hydrophilic amino-derivative, NH_2-Pap. First, a conservative amino acid mutation which also occurs in the highly homologous PylRS from *M. barkeri*[10] at the equivalent position, K192R, was introduced in order to generate a second *Bsa*I restriction site. Together with the first *Bsa*I restriction site, which had been introduced into the synthetic gene for the engineered PylRS (codon-optimized for *E. coli*) by silent mutations at the amino acid positions Gly423/Leu424, this allowed convenient cassette mutagenesis and subcloning of the central coding region encompassing the amino acid substrate pocket and active site of the enzyme.

The efficiency of cotranslational Pap incorporation was evaluated by fluorescence-activated cell sorting (FACS) of *E. coli* NEBExpress(lowRF1) constructed in this study, which was transformed with the plasmid pSB15 encoding PylRS, $tRNA^{Pyl}$ and sfGFP[11], following a published procedure[12]. sfGFP carrying the amber stop codon (TAG) at the surface-exposed and structurally permissible position 39 (sfGFPa39) was used as a fluorescent reporter protein to quantify the Pap incorporation efficiency. Compared to the same construct based on the enhanced GFP, eGFPa39, which was employed previously[12], sfGFPa39 exhibited significantly higher fluorescence in the FACS measurements. The modified PylRS from above, dubbed MmPSCaaRS(BsaI), was shown already to incorporate Pap and, albeit to a lower extent, also NH_2-Pap.

NH_2-Pap was synthesized in an attempt to increase the hydrophilicity of the unsubstituted ncAA (Pap), which is poorly soluble at neutral pH and prone to precipitation in the bacterial culture medium. Nevertheless, solubilization of plain Pap was accomplished here by complex formation with β-CD under alkaline conditions at a 1:4 stoichiometric ratio (50 mM Pap and 200 mM β-CD in 200 mM NaOH). Pap itself is readily soluble at alkaline pH whereas complex formation with β-CD effectively prevented precipitation when adding the Pap stock solution to the bacterial culture (at approximately neutral pH), with a final concentration up to 1 mM. In contrast, NH_2-Pap showed much better solubility under the same culture conditions, and a 50 mM NH_2-Pap stock solution in 100 mM HCl could be directly added to the bacterial culture without significant detectable precipitation.

In the further development of a suitable aaRS enzyme, the mutation Y384F which has been described to enhance ncAA incorporation by PylRS over a wide range of substrates with extended Lys side chains[13] was introduced by site-directed mutagenesis. This led to the mutant PapRS#0, which indeed showed higher activity towards Pap (4.4-fold increase in the MFI of sfGFPa39 as determined by FACS). However, incorporation of NH_2-Pap was still lower if applying the same concentration (0.5 mM) in the culture (Suppl. Fig. S10C).

In an attempt to improve the incorporation of NH_2-Pap, a directed evolution experiment was performed. To this end, the central PylRS region encompassing residues D196–E425 (encoded by the gene fragment flanked by the pair of *Bsa*I restriction sites) was amplified by error-prone PCR (see Methods) in order to generate a mutated plasmid library. Transformants were grown in the presence of 1 mM NH_2-Pap and subjected to FACS using the fluorescence of sfGFPa39 as readout. A subpopulation with the highest fluorescence (corresponding to 25 % of the bacteria applied) was collected to exclude loss-of-function mutations, and its pooled plasmid DNA was isolated for the following cycle of error-prone PCR and selection by FACS. The resulting 2nd generation library showed strong fluorescence for a large subpopulation specifically in the presence of 1 mM NH_2-Pap (Suppl. Fig. S10A, blue line). This average fluorescence signal was comparable to the original variant PapRS#0 grown as a single clone investigated under the same conditions (grey line).

In order to isolate mutants with further increased ncAA incorporation efficiency, three cycles of positive selection were carried out, wherein the sort gate was successively raised to collect each time 1–10 % of the cells with the highest fluorescence in the presence of NH_2-Pap. After that, the bacteria were subjected to two cycles of negative selection by collecting the subpopulation with low fluorescence in the absence of the ncAA, followed by a final positive selection. When the resulting bacterial population was grown and induced in the presence of NH_2-Pap and analyzed by FACS, the signal distribution had shifted clearly towards higher fluorescence (Suppl. Fig. S10A, red line).

Consequently, the sfGFPa39 fluorescence of individual cultures from 33 clones was examined by FACS analysis and those showing the highest MFI were subjected to plasmid sequencing.

Among these clones, six different mutated sequences were identified, which could be divided into two groups (Suppl. Fig. S11A). All four clones of the first group, PapRS#1, PapRS#13, PapRS#20, and PapRS#33, shared the amino acid substitution V346A, the only mutated residue located within the active site of PylRS. In addition, the clones PapRS#1 and PapRS#33 exhibited the mutation S221R while clone PapRS#20 carried the amino acid exchange F295L. All four clones produced similar fluorescence intensity for the substrate NH_2-Pap, which was approximately 5-fold increased compared to PapRS#0. Apparently, some additional scattered mutations present in these clones had no significant influence (Suppl. Fig. S11A). The two clones of the second group, PapRS#18 and PapRS#31, also both carried the mutation F295L and, furthermore, N304S (Suppl. Fig. S11A). NH_2-Pap incorporation was equally improved for these clones, yet to a lower extent than for those in the first group.
Interestingly, incorporation of the unsubstituted ncAA Pap was strongly enhanced for clone PapRS#31 from the second group in comparison with PapRS#0 and highest among all selected mutants, even in relation to PapRS#1 from the first group. Clone PapRS#18 from the second group, which carried the amino acid exchange S221R as seen for PapRS#1 and PapRS#33, only showed improved incorporation of NH_2-Pap but no better activity towards Pap if compared with PapRS#0 (Suppl. Fig. S10B).
Therefore, we tested whether a combination of the two groups of mutations could lead to a synergistic effect. The sequences of clones PapRS#18 and PapRS#31 as well as of the original version PapRS#0 were each combined with clones PapRS#1, PapRS#20 and PapRS#33 using a *Bam*HI restriction site that cleaves within the central coding region at the amino acid positions P317/D318, together with the *Sac*I site, leading to in total seven combination mutants (PapRS#34–40). Of these, PapRS#40 harbored V346A as the only additional mutation in relation to PapRS#0. Interestingly, this single amino acid exchange was not sufficient to reach the same level of NH_2-Pap incorporation as for the mutants of the first group discussed above (e.g. clone PapRS#1; cf. Suppl. Fig. S10C). However, the combination of the amino acid exchanges V346A with both N304S and F295L in the new mutant PapRS#34 led to a synergistic effect, resulting in higher sfGFPa39 fluorescence with the Pap substrate than for all of the previously selected clones (Suppl. Fig. S10C). Thus, PapRS#34, which carries the minimal set of mutations (K192R, F295L, A302T, N304S, L309S, N346A, C348G, Y384F) compared with the wild-type PylRS, was chosen for further studies, in particular for the preparative expression of POIs with Pap incorporated as part of the Azo-tag.
Considering the potential impact of the engineered PylRS on the bacterial fitness, it was tested whether an inducible promoter could provide an advantage over the constitutive expression as with the vector pSB15 used so far. On pSB19, the *pro*S promoter upstream of the engineered PylRS gene was replaced by the *ara*BAD$^{p/o}$[14], thus allowing induction with arabinose. Furthermore, the target gene (sfGFPa39) was placed under control of the *lac*UV5$^{p/o}$[15] instead of the *tet*$^{p/o}$[16] to avoid the use of the inducer anhydrotetracycline with its weak antibiotic activity. FACS measurements with both vectors encoding PapRS#34 revealed that the MFI was roughly double for pSB19 compared to pSB15 (Suppl. Fig. S10D). This positive effect was observed for both Pap and NH_2-Pap as ncAA substrates. In addition, bacterial cell densities were consistently higher for pSB19 at the time of harvest than with pSB15.
The benefit of the evolved aaRS (PapRS#34) in combination with the modified vector (pSB19) became even more evident when the relation between Pap concentration in the culture medium and the measured MFI was investigated. sfGFPa39 fluorescence in the FACS measurements reached saturation at a low concentration of ≤0.1 mM Pap (Fig. 3E) whereas in the case of pSB15-PapRS#0 the MFI decreased markedly at Pap concentrations already lower than 1 mM. Thus, with the expression system pSB19-PapRS#34 both the required

concentration of Pap and also the amount of the solubilizing agent β-CD was considerably reduced while still achieving better incorporation of the ncAA. Hence, this improved expression vector was used for the preparative production of various POIs carrying the Azo-tag (see Suppl. Table S1).
Of note, when using this system for the incorporation of NH_2-Pap, ESI-MS analyses of purified proteins revealed a partial reductive cleavage of the azo-bond, leading to a p-amino-L-phenylalanine (NH_2-Phe) residue at the same position, which was not observed for Pap (Suppl. Fig. S17B and C). The high specificity of PapRS#34 towards Pap as well as NH_2-Pap, together with a lack of sfGFPa39 fluorescence when supplementing the culture with NH_2-Phe for the FACS measurements, indicated that the azo-bond cleavage happens after tRNA aminoacylation. We hypothesized that the azo-bond might get reduced by the endogenous *E. coli* azo reductase (AzoR). However, the same partial cleavage was observed in a Δ*azo*R knockout strain created with the NEBExpress(lowRF1) background, leaving the mechanism of this phenomenon obscure. Therefore, further experiments focused on the use of Pap, especially as the improved incorporation efficiency with our optimized expression system required a lower concentration of this poorly soluble ncAA in the culture medium of *E. coli*.

Genome sequence analysis of NEBExpress(lowRF1)

Motivated by the observation that the presumable deletion of the *prf*A gene encoding RF-1 did not lead to 100 % amber stop codon suppression (see Suppl. Fig. S12), as should have been expected, the corresponding (unique) *E. coli* clone was subjected to further genetic analysis. First, bacterial genome sequencing employing Oxford Nanopore Technology (Eurofins Genomics, Ebersberg, Germany) with chromosomal DNA yielded contigs that exactly matched our planned genomic construct. However, when having a deeper look into the raw data of the Nanopore reads using Geneious Prime 2024.0.7 software (Biomatters, Boston, MA), we found evidence that during the genome engineering a repeated gene duplication via homologous recombination, apparently triggered by a hopping gene in the neighborhood (an insertion sequence, IS1, upstream of the *trp* operon), had occurred. Thus, the targeted *hem*A-*prf*A-*prm*C(*hem*K) operon starting with the *hem*A gene – including flanking DNA segments on both sides as well as the IS1 element to the left, in total 77.8 or 77.5 kbp – occurred in triplicate, the leftmost copy carrying the original *prf*A cistron and the middle and rightmost copies harboring the kan^r coding region instead. The single conserved copy of the *hem*A-*prf*A chromosomal region was further investigated by PCR amplification via flanking primers and Sanger dideoxy chain termination sequencing on an ABI 3730XL instrument (Eurofins Genomics), thus confirming both the absence of mutations within the reading frame for RF-1 and within the *hem*A cistron upstream, which might have led to a polar effect. Thus, the question remained why the activity of RF-1 was diminished in this genetic setting.
The *hem*A cistron encodes the enzyme glutamyl-tRNA reductase, which catalyzes the first committed step in the heme biosynthetic pathway, and whose expression is regulated both at the transcriptional and the post-translational level. In particular, it has been shown that the corresponding major promoter transcriptional start site, P1, is induced 4- to 6-fold in *E. coli* as well as in *Salmonella typhimurium* during starvation for 5-aminolevulinic acid (ALA) or heme[17]. Since *prf*A constitutes the second cistron in this operon, the expression of RF-1 is subject to the same transcriptional regulation. Considering that NEBExpress(lowRF1) carries three identical copies of the *hem*A gene – including its intact P1 promoter – two of those followed by the kan^r resistance gene but only one copy followed by the *prf*A cistron, it is conceivable that the level of the *prf*A gene product is lowered in this strain as a result of an inverse gene dosage effect.

The circumstance that we were unable to identify a mutant of NEBExpress which had the *prf*A gene simply replaced by the *kan*^{r} resistance cassette furthermore indicates that this *E. coli* B strain does not tolerate a complete loss of RF-1 – despite the absence of the activity-lowering mutation A246T in RF-2 which is common for *E. coli* K-12 strains[18]. In fact, it seems that RF-2 has to be strongly overpressed – as actually done by Johnson et al.[18] but not further explained in their publication – in order to compensate for the loss of RF-1, as it was recently demonstrated for *Salmonella enterica*[19]. Hence, it appears that the decreased but persisting expression of RF-1 in NEBExpress(lowRF1) maintains the rapid growth behavior and high recombinant protein expression capacity of the parent strain, NEBExpress, whereas the lower level of RF-1 leads to less competition at the ribosome during amber stop codon suppression by tRNA^{Pyl}. Notably, the increased yields of ncAA incorporation, by a factor up to 5 (see below), observed consistently for numerous biosynthetic proteins with the engineered *E. coli* strain NEBExpress(lowRF1) are significantly higher than the 200 % increased efficiency described for the *E. coli* K-12 strain JX33 with the presumably fully deleted *prf*A gene[18].

Enhanced amber suppression and increased expression yield of recombinant proteins having Pap incorporated as a result of partially inactivating RF-1

E. coli NEBExpress or its lowRF1 mutant was transformed with pSB19-PapRS#34 harboring the coding region for G-Pap-GG-mScarlet3. For comparison, NEBExpress(lowRF1) was transformed with pSB19-PapRS#34 encoding the mutant G-Phe-GG-mScarlet3 (using a TTT codon for Phe). Alternatively, the vector pEVOL[14] encoding AzoPheRS[20] was used for cotransformation of NEBExpress(lowRF1), together with pASK37[15] encoding G-Pap-GG-mScarlet3 under control of the *lac*UV5$^{p/o}$. The codon-optimized gene sequence for AzoPheRS was obtained by DNA synthesis (Twist Bioscience, South San Francisco, CA) and then amplified in two PCR reactions with different primer sets to consecutively replace both copies of the corresponding coding region of a different aaRS cloned on pEVOL-pAzF (Plasmid #31186; Addgene, Watertown, MA) using *Bgl*II/*Sal*I and *Nde*I/*Pst*I restriction sites, respectively. Overnight cultures were used to inoculate 500 mL LB/Amp medium (with the addition of 30 mg/L Cam in the case of pEVOL). At $OD_{550} \approx 0.4$, the culture was supplemented with 0.2 mM Pap and 0.2 % (w/v) arabinose and, 1 h later (at $OD_{550} \approx 0.8$), gene expression for the POI was induced by adding 0.5 mM IPTG. After further incubation under agitation for 18 h, the bacteria were harvested by centrifugation, and each pellet was resuspended in 10 mL SAC buffer (100 mM Tris/Cl pH 8.0, 150 mM NaCl, 1 mM EDTA), followed by cell lysis via repeated passaging through a French Pressure cell (SLM Aminco, Urbana, IL). The cell extract was cleared by centrifugation, dialyzed against SAC buffer, filter-sterilized (0.4 µm) and part of this extract, as appropriate, was subjected to protein purification on a Strep-Tactin Superflow column (5 mL bed volume, prepacked; IBA Lifesciences, Göttingen, Germany). After washing, elution was effected by applying SAC buffer supplemented with 2.5 mM D-desthiobiotin and monitored using an ÄKTA pure chromatography station with U9-D UV monitor (Cytiva) at both 280 nm and 569 nm wavelengths. Peak fractions were collected and combined, the absorbance at 280 nm was measured, and protein concentrations were calculated using extinction coefficients ε_{280} = 40910 M^{-1} cm^{-1} for G-Phe-GG-mScarlet3 (M = 27150 g/mol) and ε_{280} = 48510 M^{-1} cm^{-1} for G-Pap-GG-mScarlet3 (M = 27253 g/mol). Thus, the recombinant protein yields per liter bacterial culture were 4.2 mg for G-Pap-GG-mScarlet3 produced in NEBExpress *versus* 21.8 mg when using NEBExpress(lowRF1), indicating a 5-fold better amber suppression efficiency and high yield of purified recombinant protein from this engineered host strain. For comparison, the protein yield per liter for the conventional mutant G-Phe-GG-mScarlet3 from the same strain was 20.2 mg, which illustrates that the incorporation efficiency for the ncAA Pap, translated

from the amber stop codon, with our optimized aaRS/vector/strain system is in the same range as for a conventional codon. Finally, use of the previously described pEVOL (coexpression) vector[14], under otherwise identical conditions, resulted in a yield of 12.0 mg per liter, which was clearly inferior. Of note, pEVOL carries two identical copies of the structural gene for the AzoPheRS enzyme, even though under transriptional control of two different promoters, which was described to be crucial for efficient incorporation of Pap via amber suppression[14]. However, this also considerably complicates cloning or further mutagenesis of the engineered aaRS, apart from the effort for handling of a second expression plasmid that encodes the POI and the need for double antibiotic selection.

<u>Identification of MBP as a specific host cell contaminant in α-CD affinity chromatography</u>

Purification of POIs from periplasmic or whole cell extracts of *E. coli* via α-CD affinity chromatography showed no relevant contamination by cellular proteins except for one specific band of around 40 kDa (cf. Fig. 5A). When a total cell extract of untransformed NEBExpress(lowRF1) was loaded onto an α-CD affinity column, this contaminant was not washed off the column alongside the rest of the cellular proteins but slowly eluted over the course of more than ten column volumes in a light-independent manner (Suppl. Fig. S18B). The late wash fractions (starting after 3 mL of buffer applied) were pooled and concentrated as a homogeneous protein fraction without additional contaminations detectable by SDS-PAGE (even in the concentrated sample). The mass of the isolated protein contaminant was analyzed by ESI-MS (40706.25 Da), closely corresponding to the mass of the mature MBP, devoid of the signal peptide (calc. 40707.32 Da, UniProt ID: P0AEX9; Suppl. Fig. S18D). Its identity was further confirmed by tandem MS/MS of a trypsin-digested sample, identifying 16 peptide fragments covering 58 % of the MBP amino acid sequence. Indeed, the bound contaminant quickly eluted from an α-CD affinity column within 1.5 column volumes when washing with chromatography buffer supplemented with 50 mM maltose (Suppl. Fig. S18C). Since maltose competes with the binding of MBP to α-CD but does not bind itself to α-CD or interfere with the interaction between the ncAA Pap and α-CD, cell lysates and periplasmic extracts were supplemented with 5 mM maltose prior to loading onto the α-CD affinity column. This was sufficient to achieve high purity of Azo-tagged POIs without relevant background of cellular proteins (cf. Fig. 5B to D). As an alternative, an MBP deletion strain was generated: NEBExpress(lowRF1/ΔMBP) (cf. Fig. 3C). While its growth rate was lower than that of NEBExpress(lowRF1), cultures reached a comparable cell density. Using this new strain, POIs can be isolated from a whole cell extract in high purity with no significant cellular contaminations, thus avoiding maltose addition, as shown here for sfGFP with an N-terminal Azo-tag (Suppl. Fig. S18E and F).

<u>The role of flanking amino acids to Pap within the Azo-tag as probed using synthetic peptides in the SPOT assay and α-CD affinity chromatography</u>

An array of trimer peptides, Xaa-Pap-Yaa (324 peptides in total), with Xaa and Yaa representing all natural amino acids except for the bulky Trp and the chemically reactive Cys, was synthesized using the SPOT method and probed with an α-CD-PhoA conjugate, followed by chromogenic reaction (Suppl. Fig. S19). The signal resulting for the tripeptide sequence that had been initially fused with recombinant POIs, Ala-Pap-Gly, was only slightly above the median (2.4×10^4 versus 1.6×10^4 a.u.). However, among others, there were two tripeptides that produced more elevated signals in the range of $3.7–4.1\times10^4$ a.u.: Gly-Pap-Gly and Ser-Pap-Pro. Since in both cases the first residue has a small side chain, these sequences would still allow efficient intracellular proteolytic cleavage of the start Met or of a signal peptide.

To test the performance of the different peptide sequences in α-CD affinity purification, the corresponding variants of mScarlet3 with N-terminal Azo-tag were generated and equivalent amounts of the bacterial total cell extract (1 mL) were loaded onto the α-CD affinity column (1 mL bed volume) using the same setup as for the other purification experiments (see text). Indeed, differences in binding of the colored Azo-tagged protein to the resin became visible during the chromatography (Suppl. Fig. S20). After washing with three bed volumes of chromatography buffer A, the Gly-Pap-Gly variant still remained concentrated within the upper half of the column, whereas the initial Ala-Pap-Gly variant was already somewhat mobilized, moving slowly towards the bottom of the column with the buffer flow. Interestingly, despite exhibiting the highest signal in the SPOT screen, the Ser-Pap-Pro sequence clearly showed reduced affinity to the column. This indicates that further interactions, for example with the enzyme moiety of the α-CD-PhoA conjugate used as affinity probe, may have played a role in the SPOT assay.

The chromatographic behavior of the Gly-Pap-Gly and Ala-Pap-Gly protein versions was further compared by quantifying the fluorescence of the wash fractions after loading of the corresponding cell extracts onto the α-CD affinity column (Suppl. Fig. S20B). For the Ala-Pap-Gly sequence (red line) a certain degree of protein leakage was observed during later washing steps. In contrast, only a weakly ascending fluorescence signal for the Gly-Pap-Gly-mScarlet3 version (black line) appeared during continued washing. Accordingly, in the eluate obtained after exposure to 355 nm UV light the concentration of mScarlet3 carrying the optimized Azo-tag (Gly-Pap-Gly) was considerably higher than with the Ala-Pap-Gly sequence.

<u>Influence of salt conditions in the chromatography buffer on the light-controlled α-CD affinity chromatography of Azo-tagged proteins</u>

The interaction between the Azo-tag and immobilized α-CD appeared stable across a wide range of buffer conditions. For example, the effect of substituting 150 mM NaCl by 100 mM K_2SO_4 in the running buffer (i.e., chromatography buffer A *versus* B) on the α-CD affinity chromatography was investigated for mScarlet and Azurin, both carrying the C-terminal GG-Pap sequence (Suppl. Fig. S24). To this end, 0.5 mg (250 µL) protein pre-purified by Strep-Tactin affinity chromatography was loaded onto the α-CD affinity column (1 mL bed volume) using the same setup as for the other purification experiments (see text). In the presence of K_2SO_4, the Azo-tagged Azurin was concentrated somewhat more densely at the top of the column in comparison with the NaCl buffer. In the case of the Azo-tagged mScarlet, the fluorescence of consecutive 0.5 mL fractions, 6 mL in total, collected during the chromatography was determined. The signal in these extended washing fractions was higher with the NaCl buffer than with the K_2SO_4 buffer, and the amount of mScarlet in the eluate obtained after exposure to 355 nm UV light was significantly lower. Thus, it seems that an elevated ionic strength of the buffer can lead to improved retention and yield of an Azo-tagged POI eluted during α-CD affinity chromatography even after extensive washing. On the other hand, K_2SO_4 did not seem to increase the unspecific binding of host cell proteins to the α-CD affinity matrix. The purification of the different Azo-tagged mScarlet3 proteins shown in Fig. 5C and D was also carried out using the chromatography buffer B and did not show noticeably higher background in the eluate compared to the other purification runs. Finally, light-controlled α-CD affinity chromatography of mScarlet-*Strep*-GG-Pap was performed in the presence of the zwitterionic osmolyte trimethylamine N-oxide, TMAO[21] (50 mM Tris pH 8.0, 0.5 M TMAO), which improved the retention of the Azo-tagged POI on the α-CD affinity column compared with the neutral salt NaCl (chromatography buffer A), yet to a lesser extent than K_2SO_4 (chromatography buffer B).

Supplementary References

1 Royes, J. *et al.* Quantitative kinetic modeling in photoresponsive supramolecular chemistry: the case of water-soluble azobenzene/cyclodextrin complexes. *J Org Chem* **85**, 6509-6518 (2020).
2 Rickhoff, J. *et al.* Reversible, red-shifted photoisomerization in protonated azobenzenes. *J Org Chem* **87**, 10605-10612 (2022).
3 Jumper, J. *et al.* Highly accurate protein structure prediction with AlphaFold. *Nature* **596**, 583-589 (2021).
4 Yanagisawa, T. *et al.* Crystallographic studies on multiple conformational states of active-site loops in pyrrolysyl-tRNA synthetase. *J Mol Biol* **378**, 634-652 (2008).
5 Schmitz, K. R., Bagchi, A., Roovers, R. C., van Bergen en Henegouwen, P. M. & Ferguson, K. M. Structural evaluation of EGFR inhibition mechanisms for nanobodies/VHH domains. *Structure* **21**, 1214-1224 (2013).
6 Vaneycken, I. *et al.* Preclinical screening of anti-HER2 nanobodies for molecular imaging of breast cancer. *FASEB J* **25**, 2433-2446 (2011).
7 Voss, S. & Skerra, A. Mutagenesis of a flexible loop in streptavidin leads to higher affinity for the *Strep*-tag II peptide and improved performance in recombinant protein purification. *Protein Eng* **10**, 975-982 (1997).
8 Frank, R. The SPOT-synthesis technique: synthetic peptide arrays on membrane supports – principles and applications. *J Immunol Methods* **267**, 13-26 (2002).
9 Hoppmann, C. *et al.* Genetically encoding photoswitchable click amino acids in *Escherichia coli* and mammalian cells. *Angew Chem Int Ed Engl* **53**, 3932-3936 (2014).
10 Polycarpo, C. *et al.* Activation of the pyrrolysine suppressor tRNA requires formation of a ternary complex with class I and class II lysyl-tRNA synthetases. *Mol Cell* **12**, 287-294 (2003).
11 Pédelacq, J.-D., Cabantous, S., Tran, T., Terwilliger, T. C. & Waldo, G. S. Engineering and characterization of a superfolder green fluorescent protein. *Nat Biotechnol* **24**, 79-88 (2006).
12 Kuhn, S. M., Rubini, M., Fuhrmann, M., Theobald, I. & Skerra, A. Engineering of an orthogonal aminoacyl-tRNA synthetase for efficient incorporation of the non-natural amino acid O-methyl-L-tyrosine using fluorescence-based bacterial cell sorting. *J Mol Biol* **404**, 70-87 (2010).
13 Yanagisawa, T. *et al.* Multistep engineering of pyrrolysyl-tRNA synthetase to genetically encode N^{ε}-(o-azidobenzyloxycarbonyl) lysine for site-specific protein modification. *Chem Biol* **15**, 1187-1197 (2008).
14 Young, T. S., Ahmad, I., Yin, J. A. & Schultz, P. G. An enhanced system for unnatural amino acid mutagenesis in *E. coli*. *J Mol Biol* **395**, 361-374 (2010).
15 Skerra, A., Pfitzinger, I. & Plückthun, A. The functional expression of antibody F_V fragments in *Escherichia coli*: improved vectors and a generally applicable purification technique. *Bio/Technology* **9**, 273-278 (1991).
16 Skerra, A. Use of the tetracycline promoter for the tightly regulated production of a murine antibody fragment in *Escherichia coli*. *Gene* **151**, 131-135 (1994).
17 Choi, P., Wang, L., Archer, C. D. & Elliott, T. Transcription of the glutamyl-tRNA reductase (*hemA*) gene in *Salmonella typhimurium* and *Escherichia coli*: role of the *hemA* P1 promoter and the *arcA* gene product. *J Bacteriol* **178**, 638-646 (1996).
18 Johnson, D. B. *et al.* RF1 knockout allows ribosomal incorporation of unnatural amino acids at multiple sites. *Nat Chem Biol* **7**, 779-786 (2011).

19 Abdalaal, H., Pundir, S., Ge, X., Sanyal, S. & Näsvall, J. Collateral toxicity limits the evolution of bacterial release factor 2 toward total omnipotence. *Mol Biol Evol* **37**, 2918-2930 (2020).
20 Bose, M., Groff, D., Xie, J., Brustad, E. & Schultz, P. G. The incorporation of a photoisomerizable amino acid into proteins in *E. coli*. *J Am Chem Soc* **128**, 388-389 (2006).
21 Zou, Q., Bennion, B. J., Daggett, V. & Murphy, K. P. The molecular mechanism of stabilization of proteins by TMAO and its ability to counteract the effects of urea. *J Am Chem Soc* **124**, 1192-1202 (2002).
