# Supplementary material for: Protein purification with light via a genetically encoded azobenzene side chain
Source: Nat Commun. 2024 Dec 18;15:10693. doi: 10.1038/s41467-024-55212-y (PMC11655525; doi:10.1038/s41467-024-55212-y)
Supplement: Supplementary file 2 — Description of Additional Supplementary Files [file 41467_2024_55212_MOESM2_ESM.docx]

**Description of Additional Supplementary Files**

File Name: Supplementary_Movie_1

Description: (“Supplementary_Movie_1.mp4”): Separation of a protein mixture comprising Azurin-Strep-GG-Pap (GG-Pap alias Azo-tag) and mScarlet-Strep (without the Azo-tag) on an α-CD affinity column. While mScarlet-Strep (magenta) is washed out with the buffer flow, Azurin carrying the Azo-tag (blue) is retained at the top of the column. Subsequently, Azurin elution in the same buffer is specifically triggered by lateral exposure to 355 nm UV light. The speed of the movie has been increased, with the actual time scale shown in the top left corner (min:s).
